# Early results of PRO-EPI: PROspective multicenter observational study on elective pelvic nodes irradiation in patients with intermediate/high/very high-risk non-metastatic prostate cancer submitted to radical, adjuvant, or salvage radiotherapy with or without concomitant androgen deprivation therapy

**DOI:** 10.3389/fonc.2022.951220

**Published:** 2022-11-02

**Authors:** Andrea Emanuele Guerini, Marianna Noale, Gianluca Mortellaro, Roberto Lisi, Alessio Bruni, Roberto Santini, Paolo Muto, Giuseppe Ferrera, Gianluca Cossali, Vittorio Morelli, PRO-EPI study group, Stefano Maria Magrini, Luigi Spiazzi, Michela Buglione

**Affiliations:** ^1^ Department of Radiation Oncology, University of Brescia and Spedali Civili Hospital, Brescia, Italy; ^2^ National Research Council, Neuroscience Institute, Padova, Italy; ^3^ Department of Radiation Oncology, Azienda ospedaliera di rilievo nazionale e di alta specializzazione (ARNAS) Ospedale Civico, Palermo, Italy; ^4^ Department of Radiotherapy, Policlinico Umberto I “Sapienza” University of Rome, Rome, Italy; ^5^ Radiotherapy Unit, Oncology and Hematology Department, University Hospital of Modena, Modena, Italy; ^6^ Department of Radiation Oncology, Ospedale San Jacopo Pistoia, Pistoia, Italy; ^7^ Radiotherapy, Istituto Nazionale Tumori, “Fondazione G. Pascale”-Istituto di Ricovero e Cura a Carattere Scientifico (IRCCS), Naples, Italy; ^8^ Medical Physics Department, Azienda Socio Sanitaria Territoriale (ASST) Spedali Civili Hospital, Brescia, Italy

**Keywords:** prostate cancer, radiotherapy, pelvic nodal irradiation, ADT, IMRT (intensity modulated radiation therapy), IGRT (Image Guided Radiation Therapy), VMAT (volumetric modulated arc therapy)

## Abstract

**Simple Summary:** Although radiotherapy plays a fundamental role in the management of intermediate/high/very high-risk non-metastatic prostatic cancer (IHR-nmPca), there is still no consensus on the optimal treatment strategy in this setting. Remarkably, the role of elective nodal irradiation (ENI) is still highly controversial. The PROspective multicenter observational study on Elective Pelvic nodes Irradiation (PRO-EPI) was designed to provide “real life” data regarding the patterns of care for IHR-nmPca.

Forty-three Italian Radiation Oncology centers participated in the PROspective multicenter observational study on Elective Pelvic nodes Irradiation (PRO-EPI) project, with 1029 patients enrolled. In this preliminary analysis, we longitudinally evaluated the impact of Elective Nodal Irradiation (ENI) and radiotherapy features on toxicity and quality of life (QoL). Six months follow-up data were available for 913 patients and 12 months data for 762 patients. Elective Nodal Irradiation was given to 506 patients (48.9%). Volumetric Intensity-Modulated Radiation Therapy (IMRT) was adopted in more than 77% of patients and Image-Guided Radiation Therapy (IGRT) in 84.4%. Androgen deprivation therapy (ADT) was administered to the majority of patients (68.3%), and it was associated to ENI in 408 cases (81.1%). Toxicity was mostly mild and reversible and IGRT resulted in a significant reduction of rectal toxicity, although a non-significant trend toward increased urinary toxicity was observed. No statistically significant differences in QoL and toxicity were seen in patients treated with or without ENI. The adoption of IGRT is widespread and increasing and could reduce treatment toxicity. ENI is not yet the standard treatment, but it is performed in a growing fraction of cases and not resulting into an increase in toxicity or in a deterioration of QoL. Further analyses are needed to clarify the long-term toxicity profile and the impact of ENI on survival.

## 1 Introduction

Prostate cancer (PCa) is the second most frequently diagnosed cancer worldwide (1) and the first one in Italy accounting for 18.5% of the total new cancer cases in Italian male population, with an incidence rate of 2% in men aged older than 70 years (2). Prostate cancer presentation is extremely variable and this type of cancer affects a very heterogeneous group of patients, thus multiple treatment modalities can be offered.

Therefore, there are still many open questions regarding the optimal treatment of intermediate/high/very high-risk non-metastatic PCa (IHR-nmPca) patients, including the role of radiotherapy (3).

The most adequate treatment for IHR-nmPca, both for node positive and negative diseases and in primary and post-operative setting, still has to be defined. Controversial issues encompass the trade-off between the possible improvement of disease control and the risk of greater toxicities related to the addition of androgen deprivation therapy (ADT) and/or the inclusion of pelvic lymph nodes in treatment volume for radical or adjuvant radiotherapy (4–16).

Although radiotherapy plays a fundamental role in various setting of this disease, there is still no clear consensus regarding several aspects of its prescription and combination with systemic treatment (3).

Remarkably, the benefit of elective nodal irradiation (ENI) is debated, especially in node-negative disease and post-operative setting, although multiple analyses evaluated its potential impact (4, 5)

A systematic review published in 2014 by Dirix et al. (6) reported conflicting results, as whole pelvis radiotherapy (WPRT) improved disease-free survival (DFS) in retrospective trials, whereas the three randomized trials analyzed gave insufficient evidence to advocate the use of prophylactic ENI for IHR-nmPca.

In 2021, a new systematic review conducted by De Meerleer et al. (4), included RTOG 9413 (7), GETUG-01 (8), and the POP-RT trial (9). The POP-RT trial (9), in particular, showed improved DFS in a selected population of patients with a risk of nodal involvement greater than 20% in the group of prophylactic ENI as compared with prostate-only radiotherapy (PORT).

The adoption of ENI for the treatment of prostate cancer had no impact on overall survival (OS) in previous retrospective (13, 16) and prospective (11) studies. Coherently, an analysis of National Cancer Data Base of the United States did not show survival benefit from the addition of ENI for high-risk prostate cancer compared with PORT (12).

On the other hand, the lack of survival benefit could be due to the insufficient follow-up duration, and promising results were reported in term of biochemical progression-free survival (bPFS) in large retrospective studies evaluating ENI in combination with brachytherapy (13) or with ADT (16). In a prospective non-randomized trial by Tharmalingam et al. (11), ENI combined with brachytherapy resulted in a significant improvement in 5-year bPFS in intermediate and high-risk prostate cancer compared with PORT, regardless of ADT.

The recently published SPPORT randomized phase 3 trial (17) assessed the potential benefit of adding short-term ADT only or ENI and ADT to salvage prostate bed radiotherapy: The 5-year rate of freedom from progression improved with the addition of ADT and further increased with ADT plus ENI.

Moreover, ENI generally did not increase toxicity (16) or slightly worsened acute genitourinary and gastrointestinal toxicity (11, 15, 17), and safety profile was overall fair with no difference in late toxicity (11, 16, 17) and reported quality of life (QoL) (15).

The absence of conclusive data and strong indications might lead to relevant discrepancies across different institutions and could both deprive patients from a potentially effective treatment and lead to over-treatment and unnecessary toxicities.

Therefore, we designed a large prospective multicenter study with the aim to provide updated data on the use of ENI and ADT to treat patients with PCa undergoing elective, adjuvant, or salvage radiotherapy in Italian Radiation Oncology centers.

## 2 Materials and methods

PRO-EPI is a PROspective multicenter observational study on Elective Pelvic nodes Irradiation in patients with IHR-nmPca submitted to radical, adjuvant, or salvage radiotherapy (RT) with or without concomitant ADT.

**Figure 1:**
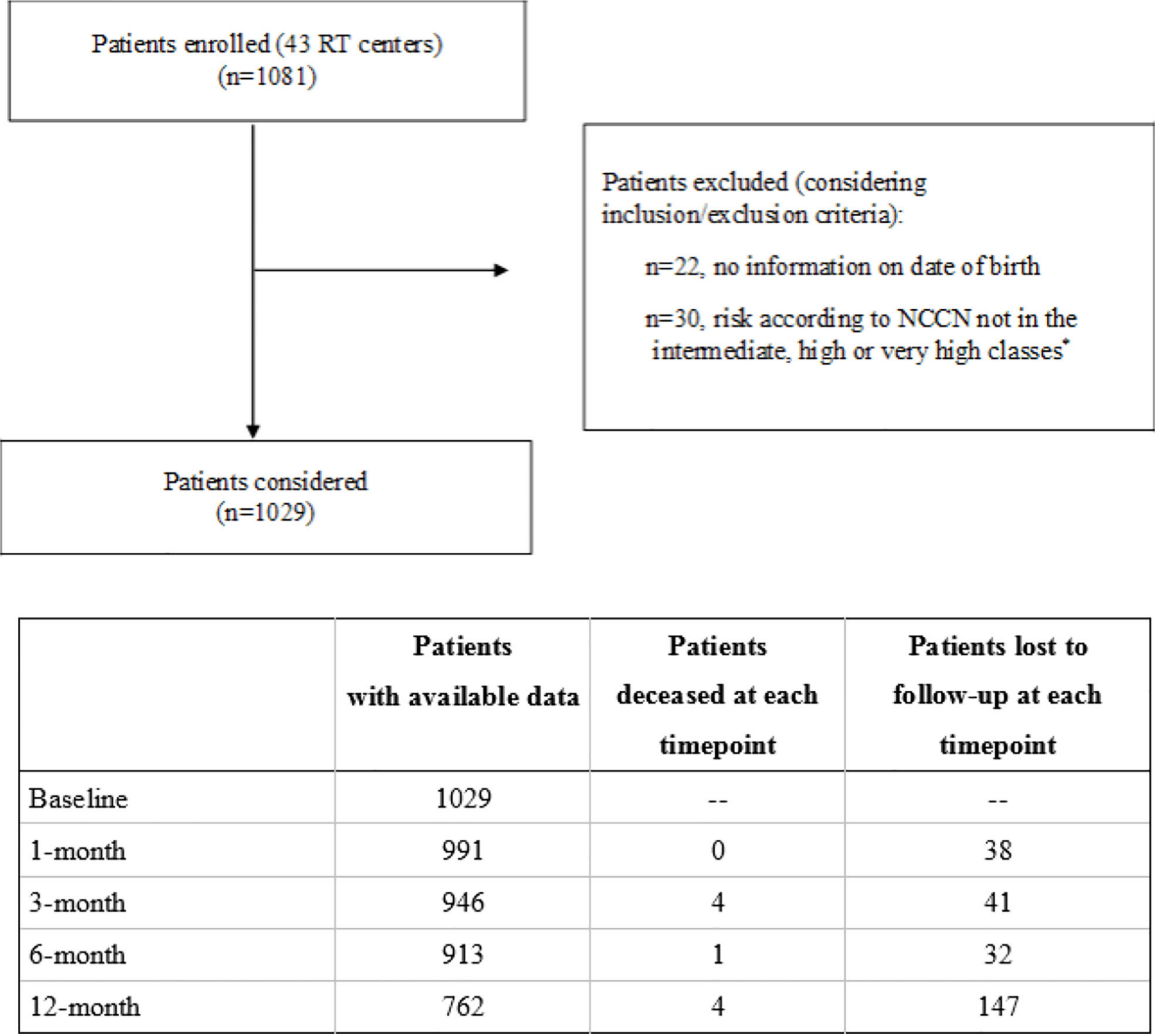
Flowchart of the PRO-EPI study. *Risk according to NCCN: intermediate (T2b, T2c, or Gleason score = 7 or 10 < PSA ≤ 20 ng/ml); high (T3a or Gleason score 8, 9, 10, or PSA > 20 ng/ml); very high (T3b, T4, or multiple risk factors for high risk).

From March 2017 to March 2020, 43 radiation oncology centers located in Italy enrolled 1,081 consecutive patients that met the inclusion criteria, as shown in **Figure 1**. Data were collected at time of enrollment and 1, 3, 6, 12, 18, 24, 30, and 36 months later.

The study was designed and carried out in accordance with the principles of the declaration of Helsinki. The study protocol was approved by the Ethics Committee of the coordinating center and by those of the other recruiting centers. All the participants signed an informed consent form.

The primary end point was OS, whereas secondary end points were cause-specific survival (CSS), biochemical relapse-free survival (bRFS), acute and late toxicity evaluation (rectal, bladder, bowel toxicity, according to common terminology criteria for adverse events [CTCAE] version 4.0 (18) and QoL assessment according to the validated Italian version of the University of California Los Angeles-Prostate Cancer Index [UCLA-PCI] and the Short-Form Health Survey Standard v1 scale (SF-12) (19, 20).

UCLA-PCI is a questionnaire of 14 multiple choice items that evaluate urinary, rectal, and sexual bother and function: Each item can have a result between zero and 100, where zero means maximum reduction of QoL and 100 means normal QoL.

SF-12 scale is a questionnaire of 12 multiple choice items that evaluate Physical Component Summary (PCS) and Mental Component Summary (MCS) through specific formulas: A higher score means better QoL.

In this preliminary analysis, the data from the first 12 months of follow-up were analyzed to evaluate the impact of ENI, ADT, and RT techniques on QoL and toxicity.

In order to compare different RT fractionations, dose to prostate and seminal vesicles was normalized to EQD2 according to this formula:

EQD2 = D (total dose given in Gy) x ([d (dose per fraction in Gy) + (α/β)]/[2 + (α/β)]).

On the basis of previous experiences (21, 22), we considered an α/β value for prostate’s tumor tissue of 1.5 Gy.

### 2.1 Statistical analysis

The categorical variables were presented as counts and percentages, whereas the continuous were summarized using means and standard deviations (*SD*s) or median and quartiles (Q1 and Q3). Normal distributions of continuous variables were tested using the Shapiro–Wilk test. Missing values were not imputed.

Differences in baseline characteristics of participants were assessed using Fisher’s exact or Chi-squared tests and Wilcoxon Rank Sum Test or generalized linear models after testing for homoschedasticity (Levene test) and for categorical and continuous variables, respectively, taking into account the following RT features: (a) aim—exclusive, adjuvant, and salvage; (b) method—image-guided radiation therapy (IGRT) *versus* no IGRT; (c) technique—three-dimensional conformal radiation therapy (3D-CRT) *versus* “step and shoot” Intensity-Modulated Radiation Therapy (IMRT) *versus* volumetric IMRT *versus* Stereotactic body radiotherapy (SBRT); (d) ENI *versus* no ENI.

Mixed-effects models were used to evaluate the changes in UCLA-PCI and SF-12 QoL scores according to ENI and RT features and time. The adjustment variables considered in the models included baseline QoL scores, age at diagnosis, comorbidities according to CIRS Comorbidity Severity Index (23, 24), presence of diabetes, and PCa risk according to NCCN. Tukey adjustments for multiple comparisons were applied.

Rectal, urinary, and bowel toxicities through follow-ups were analyzed according to RT features and ENI, considering Generalized Cochran-Mantel-Haenszel score tests of marginal homogeneity and Generalized Estimating Equations (GEEs) for ordinal repeated measures, implemented in the Genmod procedure (25) and adjusted for age at diagnosis, presence of diabetes, comorbidities according to CIRS, risk according to NCCN, and RT features.

Two-tail *p*-values < 0.05 were considered statistically significant. The analyses were performed using SAS statistical package, version 9.4 (SAS Institute Inc., Cary, NC, USA).

### 2.2 Patients and treatment characteristics

A total of 1,029 patients were enrolled; for the present analysis, 6 months follow-up data were available for 913 patients and 12 months data for 762 patients (nine died and 258 were lost to follow up, as reported in **Figure 1**).

Clinical target volume (CTV) included the prostate and the whole seminal vesicles, or the corresponding portions of the post-surgical bed, in 75.6% of cases treated with exclusive RT and 65.7% of patients receiving adjuvant RT.

The dose was prescribed with the objective to deliver more than 95% of the target prescription dose to at least 98% (D98% ≥ 95%) of each planning target volume (PTV) and less than 105% of prescribed doses to 2% of PTVs (D2% ≤ 105%).

Elective nodal irradiation was given to 503 patients (48.9%) and in more than 75% of cases (*n* = 382) the treated volumes included common iliac nodes.

## 3 Results

Comparing participants included in the analysis with those lost to follow up, the latter were older (70.1 ± 7.1 *vs*. 71.3 ± 7.2 years, respectively), whereas no significant differences were found in relation to clinical features.

### 3.1 Patients’ clinical features at enrollment

Characteristics of study participants at time of enrollment are summarized in **Table 1**. Mean age at PCa diagnosis was 70.4 ± 7.1 years (range: 36–85). It was possible to calculate the ISUP group for all the patients: 97 (9.5%) were classified as Group 1, 235 (22.8%) as Group 2, 246 (23.9%) as Group 3, 280 (27.2%) as Group 4, and 171 (16.6%) as Group 5. The majority of patients (*n* = 672, 65.3%) presented high or very high NCCN risk disease, whereas the remaining 357 patients (34.7%) presented with intermediate risk disease. Median PSA at diagnosis was 10.0 ng/ml. More than 70% of patients were diagnosed with cT2 or cT3 disease according to TNM (26), whereas 10.9% of the cases had clinical positive nodes.

**Table 1:** Characteristics of the study participants at the enrollment.

	*n* = 1029
Age at diagnosis, years	
mean ± *SD*	70.4 ± 7.1
min, max	36, 85
Education, *n* (%)	
University degree or higher High school diploma Lower secondary school diploma Elementary license None	142 (13.8) 350 (34.0) 270 (26.2) 249 (24.2) 18 (1.8)
Marital status, *n* (%)	
Married or cohabiting Widowed Separated/divorced Single	883 (85.9) 55 (5.3) 56 (5.4) 35 (3.4)
Diabetes mellitus, *n* (%)	248 (24.1)
CIRS-Comorbidity Index, median (Q1, Q3)	1 (0, 2)
CIRS-Severity Index, median (Q1, Q3)	1.2 (1.1, 1.5)
PSA at diagnosis, ng/ml, median (Q1, Q3)	10.0 (6.5, 16.6)
ISUP grade, *n* (%)	
1 2 3 4 5	97 (9.5) 235 (22.8) 246 (23.9) 280 (27.2) 171 (16.6)
Risk class, *n* (%)	
Intermediate High Very high	357 (34.7) 524 (50.9) 148 (14.4)
cT staging at diagnosis, *n* (%)	
T1 T2 T3 T4 Missing values	277 (26.9) 414 (40.2) 325 (31.6) 9 (0.9) 4 (0.4)
cN staging at diagnosis, *n* (%)	
N0 N1 NX	720 (70.0) 112 (10.9) 197 (19.1)
SF-12 PCS, mean ± SD	49.5 ± 8.0
SF-12 MCS, mean ± SD	49.9 ± 9.6
UCLA-PCI UF, mean ± SD	80.6 ± 26.9
UCLA-PCI UB, mean ± SD	75.4 ± 31.3
UCLA-PCI BF, mean ± *SD*	90.6 ± 17.0
UCLA-PCI BB, mean ± *SD*	53.0 ± 32.1
UCLA-PCI SF, mean ± *SD*	17.6 ± 27.3
UCLA-PCI SB, mean ± *SD*	87.3 ± 24.7

CIRS, Cumulative Illness Rating Scale; PSA, Prostate Specific Antigen; SD, Standard Deviation; Q1, Quartile 1; Q3, Quartile 3; UCLA-PCI, UCLA Prostate Cancer Index; UF, Urinary Function; UB, Urinary Bother; BF, Bowel Function; BB, Bowel Bother; SF, Sexual Function; SB, Sexual Bother; SF-12, Short Form survey 12; PCS, Physical Component Summary; MCS, Mental Component Summary. Scores for SF-12 PCS and MCS, and for UCLA-PCI UF, UB, BF, BB, SF, and SB range from 0 to 100, with higher scores representing better quality of life.

A comprehensive representation of patients’ features, including basal urinary, bowel and sexual function or bother and Cumulative Illness Rating Scale (CIRS) is reported in **Table 1**. Characteristics of the study participants by treatment are described in **Supplementary Tables 1 and 2** (**Supplementary Material**).

### 3.2 Treatment features

A comprehensive representation of treatment features is reported in **Table 2**.

**Table 2:** Radiotherapy and hormone therapy features.

	*n* = 1029
Aim of RT, *n* (%)	
Exclusive RT Adjuvant RT (performed within 6 months from surgery) Salvage RT (after surgery)	664 (64.6) 309 (30.0) 56 (5.4)
RT method, *n* (%)	
IGRT No IGRT Missing values	868 (84.4) 121 (11.7) 40 (3.9)
RT technique, n (%)	
IMRT (step and shoot) or 3D-CRT IMRT (volumetric) SBRT Not specified	181 (17.5) 800 (77.8) 8 (0.8) 40 (3.9)
Elective Nodal Irradiation, *n* (%)	
ENI ENI including common iliac nodes ENI not including common iliac nodes NO ENI	503 (48.9) 382 (75.9) 121 (24.1) 526 (51.1)
ADT, *n* (%) Type of ADT, *n* (%) Total androgenic blockade Androgen receptor antagonists Luteinizing hormone-releasing hormone (LH-RH) agonists LH-RH antagonists Other Not specified	703 (68.3) 69 (9.8) 55 (7.8) 494 (70.4) 77 (11.0) 1 (0.1) 7 (0.9)
Association of RT with ADT, *n* (%)	
RT without ADT RT + neoadjuvant ADT (before RT) RT + adjuvant ADT (after RT) RT + neoadjuvant + adjuvant ADT Not specified	288 (28.0) 111 (10.8) 32 (3.1) 560 (54.4) 38 (3.7)
Association of RT with ADT, *n* (%)	
ENI group NO ENI group	408/503 (81.1%) 295/526 (56.1%)

ADT, Androgen Deprivation Therapy; ENI, Elective Nodal Irradiation; IGRT, Image-Guided Radiation Therapy; IMRT, Intensity-Modulated Radiation Therapy; LH-RH, Luteinizing Hormone Releasing Hormone; SBRT, Stereotactic body radiotherapy; RT, Radiotherapy; 3D-CRT, three-dimensional conformal radiotherapy.

The majority of patients (*n* = 664, 64.6%) underwent exclusive RT, whereas 30% (*n* = 309) received adjuvant RT and 5.4% (*n* = 56) salvage RT.

More than 77% (*n* = 800) of patients were treated with volumetric IMRT and IGRT was adopted in 84.4% (*n* = 868) of patients. Concomitant, adjuvant or neoadjuvant androgen deprivation therapy (ADT) was administered to most of the patients (*n* = 703, 68.3%) and was mainly represented by LH-RH analogues (*n* = 494, 70.4% of patients treated with ADT). Median duration of ADT was 15 months (Q1 9 months, Q3 18 months). The association of RT with ADT was significantly more frequent in the group of patients that underwent ENI, compared with patients that did not (81.1% *vs.* 56.1%, *p* < 0.0001).

### 3.3 RT dose and volumes

Mean EQD2 RT dose to prostate was significantly different depending on the aim of radiotherapy: In the group of exclusive RT 92.3% (*n* = 613) received a mean EQD2 to prostate ≥ 75 Gy, whereas the surgical bed received this dose only in 97 patients (31.4%) in the group of adjuvant RT and in 19 cases in the salvage RT group (33.9%).

Most of the treatments were performed with hypofractionated schedules (>2 Gy/fraction) (*n* = 680, 66.0%). IGRT was associated with a Hypofractionated dose fractionation schedule in 72.5% of cases (*n* = 629) (*vs.* no IGRT+Hypofractionated *n* = 36, 29.8%).

Complete RT dose and volumes features are reported in **Table 3**.

**Table 3:** Radiotherapy dose and volumes.

	Aim of RT			*p*-value
	Exclusive RT (*n* = 664)	Adjuvant RT (*n* = 309)	Salvage RT (*n* = 56)	
Prostate *				< 0.0001 a ^b^ < 0.0001
EQD2 mean ± *SD* (Gy) EQD2 < 70 Gy EQD2 70-75 Gy EQD2 ≥75 Gy	79.3 ± 5.0 9 (1.4) 42 (6.3) 613 (92.3)	72.3 ± 6.7 97 (31.4) 115 (37.2) 97 (31.4)	73.6 ± 4.0 10 (17.9) 27 (48.2) 19 (33.9)	
Caudal portion of the seminal vesicles (CP) *				0.6359 < 0.0001
EQD2 mean ± *SD* (Gy) EQD2 < 70 Gy EQD2 70–75 Gy EQD2 ≥ 75 Gy Not included	73.8 ± 8.8 207 (31.2) 45 (6.8) 352 (53.0) 60 (9.0)	72.5 ± 6.8 63 (20.4) 78 (25.2) 73 (23.7) 95 (30.7)	71.5 ± 6.8 4 (7.1) 7 (12.5) 4 (7.1) 41 (73.3)	
Seminal Vesicles (SV) *				0.0001 ^a^ < 0.0001
EQD2 mean ± *SD* (Gy) EQD2 < 70 Gy EQD2 70–75 Gy EQD2 ≥ 75 Gy Not included	69.2 ± 9.1 282 (42.4) 58 (8.8) 162 (24.4) 162 (24.4)	72.2 ± 7.7 58 (18.8) 76 (24.5) 69 (22.5) 106 (34.2)	68.7 ± 6.9 7 (12.5) 5 (8.9) 3 (5.4) 41 (73.2)	
CTV including				< 0.0001
Prostate only * Prostate + CP * Prostate + CP + SV *	61 (9.2) 101 (15.2) 502 (75.6)	95 (30.7) 11 (3.6) 203 (65.7)	41 (73.2) 0 (0) 15 (26.8)	

A significant difference (p < 0.05) exclusive RT versus adjuvant RT; b significant difference (p < 0.05) exclusive RT versus salvage RT; c significant difference (p < 0.05) adjuvant RT versus salvage RT; * or the corresponding portions of the post-surgical bed. CTV, clinical target volume; SD, standard deviation.

### 3.4 Elective nodal irradiation

**Figure 2:**
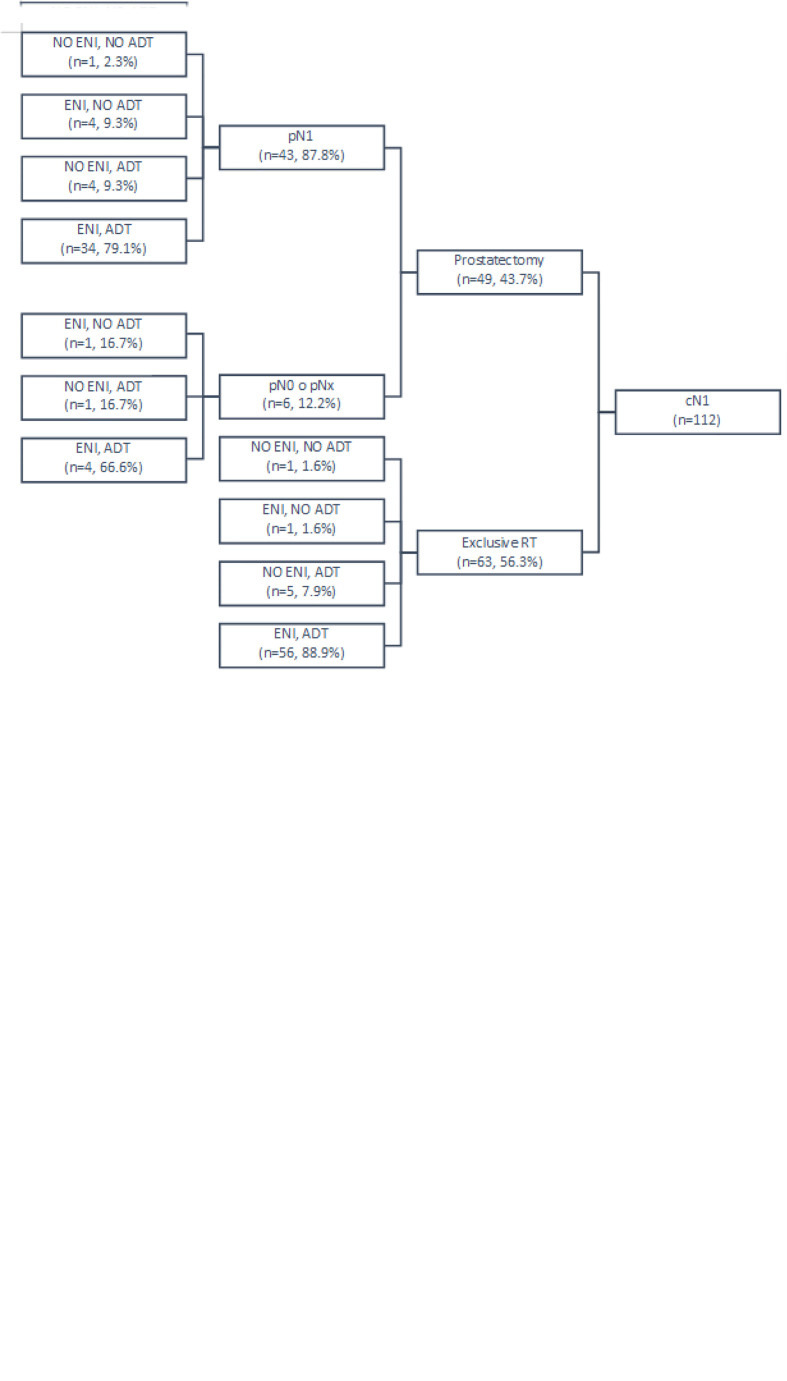
Treatment flow diagram according to nodal status.

Complete ENI features and a treatment flow diagram are reported in **Table 2** and **Figure 2**. Median prescribed dose for ENI was 50.4 Gy and median number of fractions was 28. Dose prescription for ENI was heterogeneous: 50.4 Gy was the most commonly prescribed dose (*n* = 155; 30.8%) followed by 50 Gy (*n* = 91; 18, 1%), 45 Gy (*n* = 79; 15.7%), 54 Gy (*n* = 49; 9.7%) and 56 Gy (*n* = 32; 6.4%), whereas the other 19.3% had a different prescription dose. Dose per fraction was as well variable: Most patients received 1.8 Gy/fraction (*n* = 289; 57.5%), whereas 14.9% (*n* = 75) received 2Gy/fraction, 8.5% 1.7 Gy/fraction (*n* = 43) and the remaining 19.1% a different dose per fraction.

In patients who underwent ENI, the mean dose of RT to the lymph node regions ranged from a minimum of 50.0 ± 6.9 Gy (obturator lymph nodes) to a maximum of 51.0 ± 4.4 Gy (common iliac lymph nodes). Elective nodal irradiation was performed in 100 of the 112 patients who presented with clinically positive nodes (89.3%) and 100 of the 117 patients (85.5%) with pathological positive nodes.

Androgen deprivation therapy was associated to ENI in 408 cases (81.1%). Patients treated with ENI were younger at diagnosis and had higher median PSA at diagnosis (11.5 ng/ml *vs.* 8.1 ng/ml, *p* < 0.0001). ENI was given to 405 patients with high or very high NCCN risk disease (80.5%) and 287 patients with ISUP grades 4 or 5 (57.1%).

### 3.5 Characteristics of the patients according to RT features

Patients treated with exclusive RT had a higher mean age at diagnosis, lower educational status, and a worse CIRS Comorbidity index compared with patients from other groups.

High and very high-risk disease (according to NCCN classification) and ISUP grade >2 were more frequent in the adjuvant RT group, whereas cT3 and cT4 disease were more commonly observed in patients treated post-operatively with both adjuvant or salvage RT.

Patients treated with exclusive RT presented also better basal mean scores of UCLA-PCI Urinary Function (UF), Urinary Bother (UB), Bowel Function (BF), Bowel Bother (BB), and Sexual Function (SF) compared with patients treated with post-surgical RT.

### 3.6 Quality of life

As shown in **Table 4**, **Supplementary Tables 3A, B** (**Supplementary Material**), QoL was assessed by UCLA-PCI and SF-12 at each time point (1, 3, 6, and 12 months). Comparing variation of UCLA-PCI and SF-12 scores over time, mixed models for repeated measures did not show statistically significant differences between patients that received ENI and patients that did not.

**Table 4:** Comparison of variation of UCLA-PCI and SF-12 scores over time, for ENI *versus* no ENI groups (numbers indicate estimated mean difference and 95% CI).

	Estimated differences *within groups*				Estimated differences *between groups*			*p*-value interaction group*time
	ENI	*p*-value	No ENI	*p*-value		ENI *vs.* no ENI	*p*-value	
UCLA-PCI UF								0.4919
					Baseline	0.53 (1.28)	0.9999	
1 month *vs.* baseline	-4.14 (0.95)	0.0004	-2.06 (0.84)	0.2121	1 month	-0.99 (1.46)	0.9976	
3 months *vs.* baseline	-3.58 (1.07)	0.0188	-2.54 (0.97)	0.1551	3 months	-0.42 (0.45)	0.9956	
6 months *vs.* baseline	-0.48 (0.79)	0.9988	-3.40 (1.07)	0.0329	6 months	-0.47 (0.46)	0.9906	
12 months *vs.* baseline	-1.33 (0.99)	0.8773	-2.59 (1.33)	0.5192	12 months	0.13 (0.50)	1.0000	
UCLA-PCI UB								0.3181
					Baseline	0.95 (1.87)	0.9996	
1 month *vs.* baseline	-9.36 (1.37)	< 0.0001	-4.56 (1.27)	0.0080	1 month	-3.14 (1.92)	0.7258	
3 months *vs.* baseline	-8.65 (1.51)	< 0.0001	-6.46 (1.41)	0.0001	3 months	-0.42 (0.45)	0.9956	
6 months *vs.* baseline	-1.90 (1.18)	0.7464	-8.25 (1.51)	< 0.0001	6 months	-0.47 (0.46)	0.9906	
12 months *vs.* baseline	-3.69 (1.36)	0.1178	-5.51 (1.85)	0.0597	12 months	0.13 (0.50)	1.0000	
UCLA-PCI BF								0.9311
					Baseline	-0.32 (1.35)	1.0000	
1 month *vs.* baseline	-5.53 (1.05)	< 0.0001	-4.73 (1.00)	< 0.0001	1 month	-1.63 (1.19)	0.8717	
3 months *vs.* baseline	-6.05 (1.09)	< 0.0001	-5.99 (1.08)	< 0.0001	3 months	-0.42 (0.45)	0.9956	
6 months *vs.* baseline	-1.26 (0.84)	0.8067	-6.17 (1.10)	< 0.0001	6 months	-0.47 (0.46)	0.9906	
12 months *vs.* baseline	-1.44 (0.95)	0.7994	-4.42 (1.26)	0.0114	12 months	0.13 (0.50)	1.0000	
UCLA-PCI BB								0.8140
					Baseline	2.66 (1.93)	0.8664	
1 month *vs.* baseline	-3.41 (1.5)	0.3142	-1.66 (1.43)	0.9412	1 month	1.27 (2.25)	0.9992	
3 months *vs.* baseline	-3.05 (1.75)	0.6600	-1.42 (1.57)	0.9852	3 months	-0.42 (0.45)	0.9956	
6 months *vs.* baseline	0.24 (1.35)	1.0000	-1.83 (1.78)	0.9703	6 months	-0.47 (0.46)	0.9906	
12 months *vs.* baseline	-0.16 (1.68)	1.0000	-4.32 (2.01)	0.3832	12 months	0.13 (0.50)	1.0000	
UCLA-PCI SF								0.1760
					Baseline	0.16 (0.06)	0.0840	
1 month *vs.* baseline	0.95 (0.05)	< 0.0001	0.22 (0.06)	0.0021	1 month	-1.42 (0.06)	< 0.0001	
3 months *vs.* baseline	-1.36 (0.07)	< 0.0001	1.14 (0.05)	< 0.0001	3 months	-0.42 (0.45)	0.9956	
6 months *vs.* baseline	0.92 (0.03)	< 0.0001	-1.24 (0.07)	< 0.0001	6 months	-0.47 (0.46)	0.9906	
12 months *vs.* baseline	-1.45 (0.05)	< 0.0001	0.06 (0.05)	0.9174	12 months	0.13 (0.50)	1.0000	
UCLA-PCI SB								0.9797
					Baseline	0.58 (1.73)	1.0000	
1 month *vs.* baseline	-6.62 (1.34)	<.0001	-4.17 (1.28)	0.0248	1 month	-1.95 (1.57)	0.9185	
3 months *vs.* baseline	-6.70 (1.41)	<.0001	-5.84 (1.38)	0.0006	3 months	-0.42 (0.45)	0.9956	
6 months *vs.* baseline	-1.67 (1.04)	0.7486	-5.97 (1.43)	0.0009	6 months	-0.47 (0.46)	0.9906	
12 months *vs.* baseline	-1.80 (1.27)	0.8484	-4.75 (1.63)	0.0697	12 months	0.13 (0.50)	1.0000	
SF-12 PCS								0.7436
					Baseline	0.27 (0.44)	0.9998	
1 month *vs.* baseline	-0.39 (0.37)	0.9680	0.01 (0.32)	1.0000	1 month	-0.40 (0.45)	0.9963	
3 months *vs.* baseline	0.10 (0.43)	1.0000	-0.15 (0.38)	0.9999	3 months	-0.42 (0.45)	0.9956	
6 months *vs.* baseline	-0.16 (0.32)	0.9997	-0.18 (0.43)	0.9999	6 months	-0.47 (0.46)	0.9906	
12 months *vs.* baseline	-0.20 (0.40)	0.9997	0.28 (0.50)	0.9993	12 months	0.13 (0.50)	1.0000	
SF-12 MCS								0.2071
					Baseline	-0.97 (0.55)	0.6543	
1 month *vs.* baseline	-0.93 (0.40)	0.2901	-0.69 (0.36)	0.5338	1 month	-0.63 (0.59)	0.9630	
3 month *vs.* baseline	-0.34 (0.46)	0.9959	-0.38 (0.41)	0.9847	3 months	-0.42 (0.45)	0.9956	
6 month *vs.* baseline	0.31 (0.36)	0.9902	0.29 (0.46)	0.9985	6 months	-0.47 (0.46)	0.9906	
12 month *vs.* baseline	0.98 (0.43)	0.3030	0.28 (0.55)	0.9996	12 months	0.13 (0.50)	1.0000	

ENI, ElectiveNodalIrradiation; UCLA-PCI, UCLA Prostate Cancer Index; UF, UrinaryFunction; UB, UrinaryBother; BF, BowelFunction; BB, BowelBother; SF, SexualFunction; SB, SexualBother; SF-12, Short Form Survey 12; PCS, Physical Component Summary; MCS, Mental Component Summary.

The lack of significant difference in QoL for ENI *versus* no ENI was maintained also taking into account separately patients that underwent prostatectomy before RT and patients that did not receive previous surgery.

Estimated mean differences and 95% CI from mixed-model repeated measures analyses, adjusted for score at diagnosis, age at diagnosis, presence of diabetes mellitus, number of comorbidities according to CIRS, risk according to NCCN, aim of the RT (exclusive, adjuvant, salvage), RT method (IGRT, no IGRT), RT technique (IMRT [step and shoot or 3D-CRT], IMRT [volumetric]), and ADT.

SF-12: data available at baseline for 1,017 patients, at month 1 for 918, at month 3 for 906, at month 6 for 857, at month 12 for 682 patients.

UCLA-PCI: data for UF available at baseline for 1,015 patients, at month 1 for 916, at month 3 for 905, at month 6 for 857, and at month 12 for 681 patients. Data for UB available at baseline for 1,010 patients, at month 1 for 917, at month 3 for 901, at month 6 for 853, and at month 12 for 678 patients. Data for BF available at baseline for 1,015 patients, at month 1 for 918, at month 3 for 904, at month 6 for 857, and at month 12 for 682 patients. Data for BB available at baseline for 990 patients, at month 1 for 896, at month 3 for 883, at month 6 for 836, and at month 12 for 661 patients.

Data for SF available at baseline for 991 patients, at month 1 for 915, at month 3 for 903, at month 6 for 854, and at month 12 for 678 patients. Data for SB available at baseline for 1,013 patients, at month 1 for 915, at month 3 for 903, at month 6 for 854, and at month 12 for 678 patients.

### 3.7 Treatment toxicity

**Figure 3:**
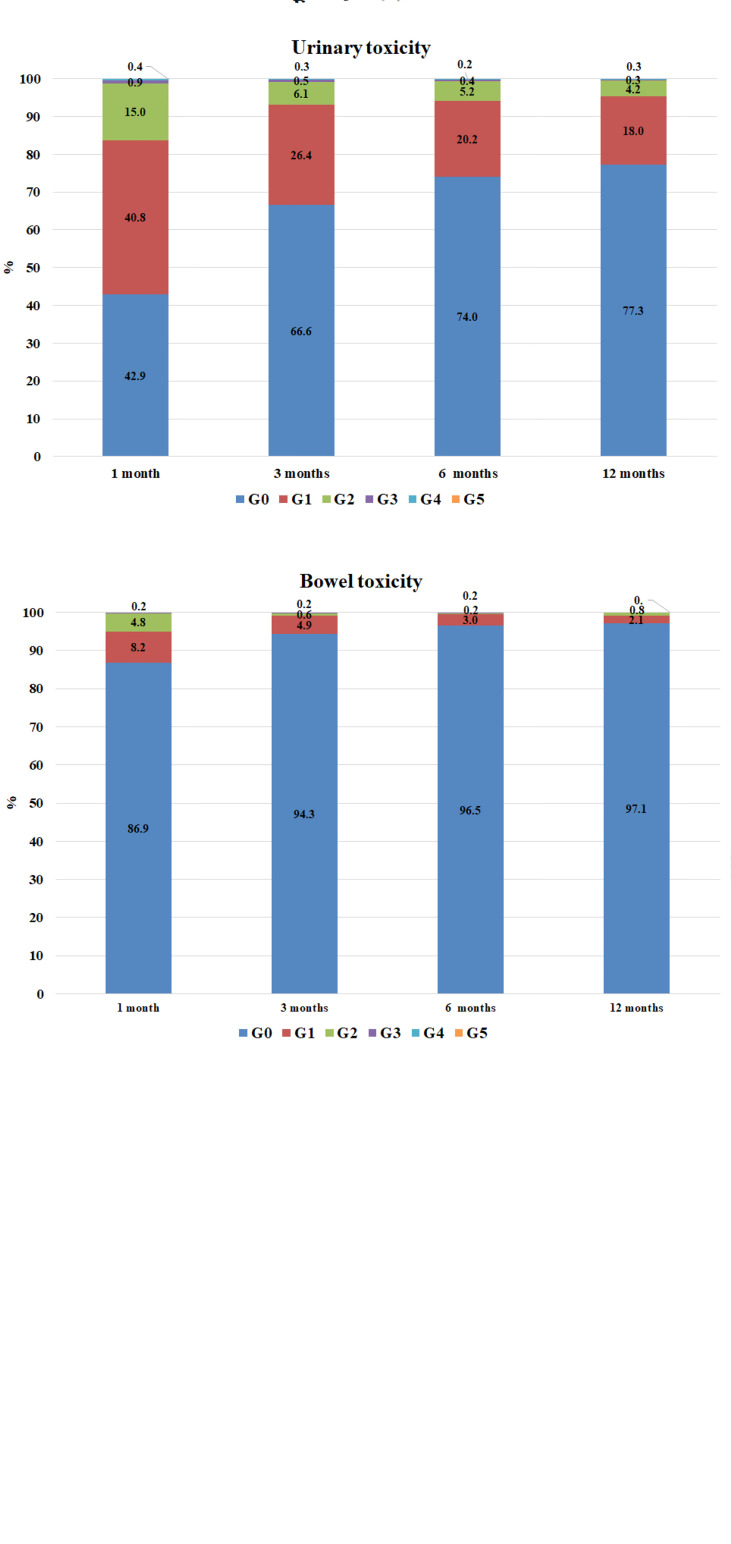
Rectal and urinary and bowel toxicity in the overall population, by time. Rectal toxicities: data available at 1 month for 990 patients, at 3 months for 965, at 6 months for 925, at 12 months for 762 patients. Urinary toxicities: data available at 1 month for 990 patients, at 3 months for 965, at 6 months for 926, and at 12 months for 762 patients.

Rectal, urinary and bowel toxicity in the overall population were classified with CTCAE v.4. A graphic representation of toxicities over time is reported in **Figure 3**.

#### 3.7.1 Rectal toxicity

At 12 months of follow-up, 73 cases of rectal toxicities were reported. These were classified according to CTCAE as G1 (*n* = 50, 68.5%), G2 (*n* = 19, 26.0%) and G3 (*n* = 4, 5.5%). Rectal toxicity was significantly more frequent in patients treated without IGRT compared with patients in IGRT group (14.4% *vs.* 8.9%, *p* = 0.0377); the odds ratio (OR) calculated with GEE for ordinal data was 0.58 (*p* = 0.0049, 95% confidence interval - CI [0.40, 0.85]) for IGRT. On the other hand, neither the aim of RT nor the technique nor ENI were associated with rectal toxicity (**Supplementary Figure 4** and **Supplementary Tables 4A, B, Supplementary Material**).

#### 3.7.2 Urinary toxicity

One hundred seventy-three cases of urinary toxicity were observed in patients with a follow-up of at least 12 months, classified as G1 (*n* = 137, 79.1%), G2 (*n* = 32, 18.5%), G3 (*n* = 2, 1.2%) and G4 (*n* = 2, 1.2%). Urinary toxicity was observed for 11.1% of patients that did not receive IGRT as opposed to 24.3% in the IGRT group (*p* = 0.0270).

The OR for urinary toxicity was of 1.41 for IGRT *versus* no IGRT (95% CI [0.98, 2.01], *p* = 0.0604). There were no statistically significant associations with ENI or RT technique, whereas previous prostatectomy correlated with urinary toxicity (OR 1.31, 95% CI 1.01–1.69, *p* = 0.0435), **Supplementary Figure 5** and **Supplementary Tables 5A, B** (**Supplementary Materials**).

#### 3.7.3 Bowel toxicity

Twenty-two cases of bowel toxicities were observed in patients followed up to at least 12 months. These were classified as G1 (*n* = 16, 72.7%) and G2 (*n* = 6, 27.3%). No cases of G3 or G4 were observed. In the group of patients not submitted to IGRT, 8.8% developed bowel toxicity versus 2.1% in the IGRT group (*p* =< 0.0001); the OR for IGRT was 0.33 (95% CI [0.19, 0.56], *p* =< 0.001).

Furthermore, bowel toxicity was more frequent in cases treated after surgery (4.8% adjuvant RT, 4.2% salvage RT) than in those submitted to exclusive RT (1.8%, *p* = 0.0310)

According to GEE for ordinal data related to bowel toxicity, there were no significant associations with the aim of RT, nor with the technique adopted, as shown in **Supplementary Figure 6** and **Supplementary Tables 6A, B** (**Supplementary Material**).

There was as well no statistically significant association between ENI and intestinal toxicity (OR 1.20, 95% CI [0.73–1.97], *p* = 0.4767).

## 4 Discussion

Up to date, there is a lack of solid data allowing to strongly recommend precautional pelvic nodal irradiation, especially in patients without clinical lymph node involvement at diagnosis (4, 5). The PRO-EPI study was designed to prospectively evaluate the current role of ENI for the treatment of patients with IHR-nmPca in a “real world” setting.

While the follow-up is still too short to draw conclusions regarding OS, CSS, and BRFS, in this preliminary analysis, we evaluated the impact of ENI, ADT, and RT techniques on QoL and toxicity.

Moreover, this large sample of patients provided a comprehensive insight into the treatment paths that are at present endorsed by multiple Italian institutions (43 centers involved), and it is hence representative of the current Italian scenario.

The first relevant data concern the growing use of IGRT and volumetric IMRT, which have been adopted in about 85 and 75% of the cases, respectively.

This finding confirms the striking and relentless evolution of RT techniques, if we consider that in the recently published POP III study, which analyzed the pattern of practice of Italian radiation oncology centers in 2004–2011 period, IGRT was used only in 13% of cases (27).

The use of ENI as well apparently increased over time; as in POP III study, it was prescribed only to 4% of cases (27), whereas in this analysis about half of the patients received prophylactic pelvic irradiation. On the other hand, this demonstrates that there is still no consensus regarding the opportunity to offer ENI to IHR-nmPca patients. From the data collected, it is also possible to deduce that Italian radiation oncologists propose ENI more often to patients with negative prognostic factors, such as a higher ISUP score, a higher clinical T or higher initial PSA level. This is in line with the recommendations derived from the recently published POP-RT study (8).

The characteristics of patients undergoing exclusive radiotherapy are similar to those already described in previous studies, such as Pros-IT (28): older, with more comorbidities and a lower level of education than patients who have previously undergone surgery.

As expected, patients undergoing adjuvant RT tended to have a higher NCCN risk class and cT3 or cT4 diseases are more represented among patients treated post-operatively.

Regarding the QoL perceived by patients, the SF-12 and UCLA questionnaires revealed that there were no significant differences in the trajectories of QoL domains up to 12 months after treatment between patients who underwent ENI and patients that did not. Of note, we reported no statistically significant difference in QoL measures between patients that received ENI *versus* patients that did not, despite ADT was administered in a larger fraction of subjects in ENI group (81.1% *vs.* 56.1%). This result was coherent with the absence of an increase in rectal, urinary, and bowel toxicity reported by the clinicians. It has to be noted that, while a larger decrease in QoL could be expected due to the concurrent use of ADT, some previous experience retrieved a limited impact of its administration on global QoL in patients with prostate cancer. For example, the CaPSURE registry enrolled 3,068 men, comparing QoL of patients that underwent prostatectomy, RT with or without ADT and ADT alone: Treatment group was not associated in clinically meaningful decrease in QoL (29). Patient-reported outcome measures (PROMs) and QoL assessment are a valuable mean to improve the communication between clinicians and patients, detect side effects and optimize therapeutic workflow and supportive care (30). Nonetheless, although questionnaires such as SF-12 have been validated for cancer patients, the self-reported nature of the data might be flawed by recall bias, remarkably for intimate topics such as sexual function (31).

Statistically significant differences were seen in QoL at the time of recruitment between patients who had previously undergone surgery and those who were candidates for exclusive RT treatment, as already reported in previous experiences (32).

Patients undergoing radical RT, compared with those previously treated with surgery, tended to have better basal scores on the questionnaires administered at the time of recruitment.

The toxicity profile was overall fair, as rates of G3–G4 adverse events were extremely low. The trend, as shown in **Figure 3**, is characterized by a higher frequency of mild and mostly urinary acute toxicity and by a subsequent progressive and gradual reduction in the severity and frequency of the side effects.

Remarkably, the group of subjects treated with ENI did not report higher rates and/or severity of adverse events compared with patients that received RT only to the prostate and seminal vesicles. In previous experiences, ENI toxicity profile was as well overall safe: While in some instances it slightly increased acute toxicity (11, 15, 17), it did not increase late toxicity (11, 16). In the SPPORT randomized phase 3 trial, adding ENI to prostate-bed RT in the salvage setting resulted in a greater rate of acute grade 2 or worse adverse events, but no significant difference was reported for late toxicity apart from increased hematologic side effects (17). Although final conclusions still could not be drawn regarding the indication of ENI, this lack of increased toxicity combined with the potential benefit in term of disease control could support its use at least in patients with clinically positive lymph nodes or at higher risk of nodal relapse. For example, in the salvage setting after surgery, addition of ENI to ADT and radiotherapy on the prostate bed resulted in higher rates of freedom from disease progression at 5 years in the phase 3 randomized SPPORT trial (17).

The adoption of IGRT resulted in a significant reduction of rectal and intestinal toxicity. On the other hand, a non-statistically significant trend toward higher urinary toxicity was reported in IGRT group. This finding could be explained by the greater use of hypofractionated RT schedule in these patients. A similar trend, with a mild statistically significant increase in late urinary toxicity in patients undergoing hypofractionated IGRT, compared with the no IGRT group, was also found in a recent study by Jereczek et al. (33).

The limits of this study must be as well acknowledged. First, this represents only a preliminary analysis, as long-term data are awaited to define the impact of different treatment modalities and techniques on clinical outcomes and late toxicities [including, e.g., the metabolic effects of ADT (34)].

The other main limit is the high rate of patients lost to follow up at 12 months (25%). In order to provide a comprehensive picture of the adoption of ENI and ADT in Italian radiation oncology centers, 43 institutions were enrolled. Unfortunately, compliance of some participants to timely transmit collected data for this preliminary analysis was sub-optimal. This highlighted the necessity of a coordinated and continuous data monitoring, given the high number of involved institutions. Dedicated measures have been taken to overcome this pitfall and reduce the number of patients lost at follow-up in the final analysis of the study.

Defining a correlation between dosimetric parameters and clinical outcomes is essential to evaluate the actual benefit of modern radiotherapy techniques such as IMRT and IGRT. In this preliminary study, information regarding the ionizing radiation dose received by the OARs was not evaluated, but we are currently collecting data to integrate a dosimetric analysis in the final results of the study. These data should be as well interpreted according to the results of multiple recent trials, suggesting that α/β ratio for prostate cancer could be higher than previously estimated (35).

Moreover, since the start of this study, modern imaging and radiotherapy techniques that can change IHR-nmPca treatment have been increasingly adopted.

Next generation imaging (including whole-body diffusion-weighted MRI and positron emission tomography), with novel radiopharmaceuticals, is increasingly used as a mean to allow optimal local staging and early identification of distant metastases (36).

Remarkably, the adoption of prostate-specific membrane antigen PET/CT (PSMA-PET/CT) is rapidly expanding due to its ability to detect nodal disease earlier than conventional imaging and at relatively lower levels of PSA (36, 37).

The recently published results of the prospective, randomized proPSMA trial (33) confirmed that PSMA-PET/CT has higher sensitivity and specificity compared with conventional imaging (CT and bone scan) both for nodal and distant metastasis in men with high-risk prostate cancer undergoing staging before curative-intent therapy.

Although, currently, PSMA-PET/CT is mostly used for re-staging the disease after biochemical recurrence (37, 38), its integration in frontline staging at first diagnosis for IHR-nmPca could provide information not detectable with conventional imaging in a large fraction of patients, which might change the management in about a third of the cases (39).

The widespread use of PSMA-PET/CT in this setting could thus improve the diagnostic process and allow to offer a more tailored treatment, for example, allowing an early identification of low-burden node positive patients that could benefit from ENI.

Emerging RT techniques, such as MR-guided radiotherapy, could further change the landscape of IHR-nmPca treatment and preliminary studies suggest promising results in term of tolerability and dosimetric results (40).

Nonetheless, it should be considered that the potential clinical benefit of these innovative imaging and radiotherapy options still has to be assessed and clarified.

## 5 Conclusions

This preliminary analysis highlighted the widespread and growing use of IGRT and volumetric IMRT in Italy for the treatment of IHR-nmPca, potentially allowing a further reduction of RT-induced toxicity.

Although there is currently no consensus regarding the indications for ENI for IHR-nmPca, its adoption seemed to increase over years as well.

In our series, offering ENI to intermediate and high-risk patients did not translate into an increase in short-term toxicity or in a deterioration of quality of life, the main concerns limiting its use. Follow-up of our series is too short to draw significant conclusions regarding the impact of different techniques, ENI and ADT on disease control and survival.

## Data availability statement

The raw data supporting the conclusions of this article will be made available by the authors, without undue reservation.

## Ethics statement

This study was reviewed and approved by Spedali Civili di Brescia Ethics Committee. The patients/participants provided their written informed consent to participate in this study.

## PRO-EPI study group

Stefano Maria Magrini, Luca Triggiani, Claudia Cozzaglio, Maurizio Valeriani, Emanuela Cagna, Giovanna Mantini, Anna Rita Alitto, Sarah Colangione, Rita Bellavita, Isabella Palumbo, Salvatore Parisi, Matteo Sepulcri, Alessandra Corsini, Simona Fondelli, Lucia Amara, Maria Tamburo, Sergio Fersino, Luigi Marafioti, Ciro Franzese, Giuseppe D’Agostino, Simona Borghesi, Salvina Barra, Gianluca Ingrosso, Lorenzo Livi, Elisa Peci, Maurizio Kalli, Andrea Galla, Angelo Errico, Mariangela La Macchia, Angelo Giuseppe Platania, Paolo Antognoni, Tindaro Scolaro, Stefano Arcangeli, Massimo Cardinali, Francesco Fenu, Serena Ciabatti, Sabino Bonaduce, Dario Zerini, Francesca Maggio, Francesca Ciriello, Elisabetta Vitali, Alessandro Magli, Alessandro Sindoni, Marcello Amadori, Vincenza Umina.

## Author contributions

Conceptualization MN, GM, RL, AB, RS, PM, PRO-EPI study group, LS and MB. Data curation MN, RL, AB, RS, PM, GF, GC, VM, PRO-EPI study group, LS and MB. Formal analysis AG, MN, GF, VM, PRO-EPI study group, LS and MB. Investigation, AG, GM, RL, AB, GF, GC, VM, PRO-EPI study group, LS and MB. Methodology, AG, GM, RL, AB, RS, PM, PRO-EPI study group, LS and MB. Project administration, RS, PM and PRO-EPI study group. Supervision, AG, MN, GM, RL, AB, RS, PM, GF, PRO-EPI study group, LS and MB. Validation, MN, GF and GC. Visualization, GC and VM. Writing – original draft, AG, MN, GM, RL, AB, RS, PM, GF, GC, VM, PRO-EPI study group, LS and M.B. Writing – review and editing, AG, MN and PRO-EPI study group. All authors contributed to the article and approved the submitted version.

## Funding

This research received non conditioning funding from Takeda

## Acknowledgments

We would like to thank Dr. Filippo Bertoni for his contribution as an advisor to the Principal Investigator and for the coordination of the study.

## Conflict of interest

The authors declare that the research was conducted in the absence of any commercial or financial relationships that could be construed as a potential conflict of interest.

## Publisher’s note

All claims expressed in this article are solely those of the authors and do not necessarily represent those of their affiliated organizations, or those of the publisher, the editors and the reviewers. Any product that may be evaluated in this article, or claim that may be made by its manufacturer, is not guaranteed or endorsed by the publisher.
