# Supplementary material for: Early results of PRO-EPI: PROspective multicenter observational study on elective pelvic nodes irradiation in patients with intermediate/high/very high-risk non-metastatic prostate cancer submitted to radical, adjuvant, or salvage radiotherapy with or without concomitant androgen deprivation therapy
Source: Front Oncol. 2022 Nov 2;12:951220. doi: 10.3389/fonc.2022.951220 (PMC9666761; doi:10.3389/fonc.2022.951220)

**Supplementary Table 1.** Characteristics of the study participants at the enrollment by ENI

|  | **ENI** | | |  |  |
| --- | --- | --- | --- | --- | --- |
|  | **no ENI**  **(n=488)** | **ENI**  **(n=503)** | **p-value** | | |
| Age at diagnosis, years, mean±SD | 71.6±6.8 | 69.2±31.1 | <0.0001 | | |
| Education, ≤lower secondary school, n (%) | 255 (52.3) | 257 (51.1) | 0.7147 | | |
| Marital status, married or cohabiting, n (%) | 425 (87.1) | 425 (84.5) | 0.2420 | | |
| Diabetes mellitus, n (%) | 123 (25.2) | 112 (22.3) | 0.2769 | | |
| CIRS-Comorbidity Index≥2 | 182 (37.3) | 148 (29.4) | 0.0086 | | |
| PSA at diagnosis, ng/ml, median (Q1, Q3) | 8.1 (5.8, 13.5) | 11.5 (7.9, 20.1) | <0.0001 | | |
| ISUP grade, n (%)  1  2  3  4  5 | 58 (11.9)  152 (31.2)  129 (26.4)  98 (20.1)  51 (10.5) | 34 (6.8)  73 (14.5)  109 (21.7)  172 (34.2)  115 (22.9) | <0.0001 | | |
| Risk class, n (%)  Intermediate  High  Very high | 245 (50.2)  194 (39.8)  49 (10.0) | 98 (19.5)  311 (61.8)  94 (18.7) | <0.0001 | | |
| cTstaging at diagnosis, n (%)  T1  T2  T3 or T4 | 118 (24.2)  229 (46.9)  141 (28.9) | 135 (26.8)  179 (35.6)  189 (37.6) | 0.0005 | | |
| SF-12 PCS, mean±SD | 50.0±7.6 | 49.1±8.4 | 0.4307 | | |
| SF-12 MCS, mean±SD | 49.2±9.2 | 50.5±9.9 | 0.0028 | | |
| UCLA-PCI UF, mean±SD | 84.9±23.9 | 76.0±29.1 | <0.0001 | | |
| UCLA-PCI UB, mean±SD | 77.6±31.0 | 72.6±31.8 | 0.0043 | | |
| UCLA-PCI BF, mean±SD | 90.5±16.5 | 90.3±17.9 | 0.4993 | | |
| UCLA-PCI BB, mean±SD | 55.3±32.5 | 50.6±31.5 | 0.0172 | | |
| UCLA-PCI SF, mean±SD | 21.2±30.1 | 14.1±23.2 | 0.0009 | | |
| UCLA-PCI SB, mean±SD | 86.7±24.7 | 87.6±25.0 | 0.3410 | | |

*Abbreviations:* CIRS, Cumulative Illness Rating Scale; ENI, Elective Nodal Irradiation; PSA, Prostate Specific Antigen; Q1, Quartile 1; Q3, Quartile 3;SD, Standard Deviation; UCLA-PCI, UCLA Prostate Cancer Index; UF, Urinary Function; UB, Urinary Bother; BF, Bowel Function; BB, Bowel Bother; SF, Sexual Function; SB, Sexual Bother; SF-12, Short Form survey 12; PCS, Physical Component Summary; MCS, Mental Component Summary.

**Supplementary Table 2.** Characteristics of the study participants at the enrollment by RT features

|  | **Aim of RT** | | | | **Method** | | |
| --- | --- | --- | --- | --- | --- | --- | --- |
|  | **Exclusive RT**  **(n=664)** | **Adjuvant RT**  **(n=309)** | **Salvage RT**  **(n=56)** | **p-value** | **no IGRT**  **(n=121)** | **IGRT**  **(n=868)** | **p-value** |
| Age at diagnosis, years, mean±SD | 73.3±5.7 | 65.3±6.6 | 65.2±6.7 | <0.0001 | 70.1±6.7 | 70.4±7.2 | 0.4038 |
| Education ≤ lower secondary school, n (%) | 381 (57.4) | 130 (42.1) | 26 (46.4) | <0.0001 | 63 (52.1) | 448 (51.6) | 0.9255 |
| Marital status, married or cohabiting, n (%) | 562 (84.6) | 272 (88.0) | 49 (87.5) | 0.3454 | 99 (81.8) | 751 (86.5) | 0.1632 |
| Diabetes mellitus, n (%) | 167 (25.2) | 71 (23.0) | 10 (17.9) | 0.4052 | 33 (27.3) | 202 (23.3) | 0.3327 |
| CIRS-Comorbidity Index≥2 | 275 (41.4) | 63 (20.4) | 9 (16.1) | <0.0001 | 58 (47.9) | 271 (31.2) | 0.0003 |
| PSA at diagnosis, ng/ml, median (Q1, Q3) | 9.9 (6.4, 16.1) | 10 (6.7, 18) | 11.5 (6.8, 18.7) | 0.4943 | 10.9 (7, 18.8) | 9.8 (6.4, 16.4) | 0.1672 |
| ISUP grade, n (%)  1  2  3  4  5 | 67 (10.1)  165 (24.9)  144 (21.7)  185 (27.9)  103 (15.5) | 21 (6.8)  56 (18.1)  89 (28.8)  84 (27.2)  59 (19.1) | 9 (16.1)  14 (25.0)  13 (23.2)  11 (19.6)  9 (16.1) | 0.0312 | 20 (16.5)  24 (19.8)  39 (32.2)  24 (19.8)  14 (11.6) | 71 (8.2)  201 (23.2)  199 (22.9)  246 (28.3)  151 (17.4) | 0.0016 |
| Risk class, n (%)  Intermediate  High  Very high | 241 (36.3)  344 (51.8)  79 (11.9) | 96 (31.1)  148 (47.9)  65 (21.0) | 20 (35.7)  32 (57.1)  4 (7.1) | 0.0018 | 50 (41.3)  52 (43.0)  19 (15.7) | 292 (33.6)  452 (52.1)  124 (14.3) | 0.1584 |
|  |  |  |  |  |  |  |  |
|  |  |  |  |  |  |  |  |
| cTstaging at diagnosis, n (%) *  T1  T2  T3, T4 | 187 (28.1)  281 (42.3)  192 (28.9) | 83 (27.0)  107 (34.7)  119 (38.4) | 7 (12.5)  26 (46.4)  23 (41.1) | 0.0135 | 17 (14.1)  63 (52.1)  41 (33.9) | 236 (27.2)  343 (39.5)  289 (33.3) | 0.0047 |
| SF-12 PCS, mean±SD | 49.1±8.2 | 49.9±7.5 | 51.9±7.7 | 0.0042 | 49.9±7.7 | 49.6±9.4 | 0.0006 |
| SF-12 MCS, mean±SD | 49.9±9.6 | 49.9±9.8 | 49.8±7.8 | 0.7749 | 50.3±9.6 | 47.3±9.4 | 0.0003 |
| UCLA-PCI UF, mean±SD | 92.7±14.4 | 56.5±29.4 | 71.1±31.6 | <0.0001 | 81.4±26.3 | 73.6±31.0 | 0.0108 |
| UCLA-PCI UB, mean±SD | 85.0±24.4 | 57.3±34.1 | 63.4±37.5 | <0.0001 | 76.4±30.7 | 65.7±35.1 | 0.0011 |
| UCLA-PCI BF, mean±SD | 91.4±15.8 | 88.3±19.8 | 93.7±13.7 | 0.0733 | 91.0±16.6 | 86.4±20.3 | 0.0102 |
| UCLA-PCI BB, mean±SD | 57.6±32.4 | 45.6±30.4 | 40.9±28.2 | <0.0001 | 54.4±32.4 | 41.9±27.5 | <0.0001 |
| UCLA-PCI SF, mean±SD | 22.7±30.4 | 8.1±16.9 | 11.8±19.5 | <0.0001 | 17.8±27.5 | 15.1±22.4 | 0.8200 |
| UCLA-PCI SB, mean±SD | 87.0±25.1 | 87.7±24.3 | 89.7±22.2 | 0.7361 | 87.6±24.5 | 84.5±26.7 | 0.0998 |
| Dose fractionation schedule  Hypofractionated (>2 Gy/fraction)  Normofractionated (1.8-2 Gy/fraction)  Hyperfractionated (<1.8 Gy/fraction) | 471 (71.0)  192 (28.9)  1 (0.1) | 165 (53.5)  139 (44.9)  5 (1.6) | 44 (78.6)  12 (21.4)  0 (0.0) | <0.0001 | 36 (29.8)  85 (70.2)  0 (0.0) | 629 (72.5)  233 (26.8)  6 (0.7) | <0.0001 |

*Abbreviations:*CIRS, Cumulative Illness Rating Scale; IGRT, Image-GuidedRadiation Therapy; PSA, Prostate SpecificAntigen; RT, Radiotherapy; Q1, Quartile 1; Q3, Quartile 3; SD, Standard Deviation; UCLA-PCI, UCLA Prostate Cancer Index; UF, UrinaryFunction; UB, UrinaryBother; BF, BowelFunction; BB, BowelBother; SF, SexualFunction; SB, SexualBother; SF-12, Short Form survey 12; PCS, Physical Component Summary; MCS, Mental Component Summary.

* Not including missing values

**Supplementary Table 3a.** Comparison of variation of UCLA-PCI and SF-12 scores over time, for ENI vs no ENI groups (numbers indicate estimated mean difference and 95% CI), considering **only patients submitted to prostatectomy**

|  | **Estimated differences**  ***within groups*** | | | |  | **Estimated differences**  ***between groups*** | | | **p-value interaction**  **group*time** |
| --- | --- | --- | --- | --- | --- | --- | --- | --- | --- |
|  | **ENI** | **p-value** | **No ENI** | **p-value** |  |  | **ENI vs no ENI** | **p-value** |  |
| UCLA-PCI UF |  |  |  |  |  |  |  |  | 0.7810 |
|  |  |  |  |  |  | Baseline | 0.29 (2.41) | 1.0000 |  |
| 1 months vs baseline | -4.77 (1.48) | 0.0294 | -2.77 (1.82) | 0.7925 |  | 1 month | -1.68 (2.95) | 0.9992 |  |
| 3 months vs baseline | -4.74 (1.86) | 0.1802 | -2.85 (2.00) | 0.8434 |  | 3 months | -0.42 (0.45) | 0.9956 |  |
| 6 months vs baseline | -0.08 (1.60) | 1.0000 | -4.61 (2.46) | 0.5723 |  | 6 months | -0.47 (0.46) | 0.9906 |  |
| 12 months vs baseline | -1.84 (2.30) | 0.9931 | -3.06 (2.67) | 0.9464 |  | 12 months | 0.13 (0.50) | 1.0000 |  |
| UCLA-PCI UB |  |  |  |  |  |  |  |  | 0.3838 |
|  |  |  |  |  |  | Baseline | 4.61 (3.23) | 0.8434 |  |
| 1 months vs baseline | -7.93 (1.84) | 0.0006 | -1.64 (2.39) | 0.9973 |  | 1 month | -0.69 (3.58) | 1.0000 |  |
| 3 months vs baseline | -6.94 (2.40) | 0.077 | -3.44 (2.50) | 0.8662 |  | 3 months | -0.42 (0.45) | 0.9956 |  |
| 6 months vs baseline | -1.80 (2.11) | 0.9899 | -6.23 (3.18) | 0.5085 |  | 6 months | -0.47 (0.46) | 0.9906 |  |
| 12 months vs baseline | -4.59 (2.85) | 0.7428 | -6.25 (3.33) | 0.5679 |  | 12 months | 0.13 (0.5) | 1.0000 |  |
| UCLA-PCI BF |  |  |  |  |  |  |  |  | 0.3409 |
|  |  |  |  |  |  | Baseline | -0.21 (2.31) | 1.0000 |  |
| 1 months vs baseline | -6.77 (1.44) | 0.0001 | -2.71 (1.92) | 0.8496 |  | 1 month | -4.31 (2.00) | 0.3793 |  |
| 3 months vs baseline | -6.81 (1.47) | 0.0001 | -5.76 (1.94) | 0.0633 |  | 3 months | -0.42 (0.45) | 0.9956 |  |
| 6 months vs baseline | -3.05 (1.39) | 0.3605 | -8.19 (1.97) | 0.0011 |  | 6 months | -0.47 (0.46) | 0.9906 |  |
| 12 months vs baseline | -5.48 (1.57) | 0.0127 | -2.50 (2.14) | 0.9405 |  | 12 months | 0.13 (0.50) | 1.0000 |  |
| UCLA-PCI BB |  |  |  |  |  |  |  |  | 0.1646 |
|  |  |  |  |  |  | Baseline | 6.18 (3.04) | 0.4598 |  |
| 1 months vs baseline | -4.30 (2.15) | 0.4812 | 3.10 (2.59) | 0.9323 |  | 1 month | 2.46 (3.50) | 0.9969 |  |
| 3 months vs baseline | -0.62 (2.36) | 1.0000 | 1.14 (2.09) | 0.9999 |  | 3 months | -0.42 (0.45) | 0.9956 |  |
| 6 months vs baseline | -1.96 (2.18) | 0.9859 | 1.25 (3.14) | 0.9999 |  | 6 months | -0.47 (0.46) | 0.9906 |  |
| 12 months vs baseline | -1.85 (2.70) | 0.9974 | -3.08 (3.23) | 0.9805 |  | 12 months | 0.13 (0.50) | 1.0000 |  |
| UCLA-PCI SF |  |  |  |  |  |  |  |  | 0.0873 |
|  |  |  |  |  |  | Baseline | 0.11 (0.09) | 0.9337 |  |
| 1 months vs baseline | 0.89 (0.07) | <0.0001 | 0.00 (0.10) | 1.0000 |  | 1 month | -1.21 (0.09) | <0.0001 |  |
| 3 months vs baseline | -1.32 (0.10) | <0.0001 | 1.06 (0.09) | <0.0001 |  | 3 months | -0.42 (0.45) | 0.9956 |  |
| 6 months vs baseline | 1.06 (0.05) | <0.0001 | -1.34 (0.13) | <0.0001 |  | 6 months | -0.47 (0.46) | 0.9906 |  |
| 12 months vs baseline | -1.34 (0.09) | <0.0001 | -0.11 (0.07) | 0.8006 |  | 12 months | 0.13 (0.50) | 1.0000 |  |
| UCLA-PCI SB |  |  |  |  |  |  |  |  | 0.2661 |
|  |  |  |  |  |  | Baseline | 2.92 (2.83) | 0.9690 |  |
| 1 months vs baseline | -7.40 (1.84) | 0.0018 | 0.37 (2.47) | 1.0000 |  | 1 month | -5.34 (2.57) | 0.4325 |  |
| 3 months vs baseline | -7.89 (1.95) | 0.0017 | -3.38 (2.48) | 0.8739 |  | 3 months | -0.42 (0.45) | 0.9956 |  |
| 6 months vs baseline | -3.75 (1.78) | 0.4106 | -6.60 (2.62) | 0.1895 |  | 6 months | -0.47 (0.46) | 0.9906 |  |
| 12 months vs baseline | -6.98 (2.35) | 0.0626 | -2.55 (2.71) | 0.9820 |  | 12 months | 0.13 (0.50) | 1.0000 |  |
| SF-12 PCS |  |  |  |  |  |  |  |  | 0.3139 |
|  |  |  |  |  |  | Baseline | 0.48 (0.82) | 0.9991 |  |
| 1 months vs baseline | -0.59 (0.55) | 0.9639 | 0.89 (0.66) | 0.8782 |  | 1 month | -0.44 (0.83) | 0.9995 |  |
| 3 months vs baseline | -0.02 (0.61) | 1.0000 | 0.52 (0.75) | 0.9969 |  | 3 months | -0.42 (0.45) | 0.9956 |  |
| 6 months vs baseline | -0.37 (0.55) | 0.9979 | 0.06 (0.81) | 1.0000 |  | 6 months | -0.47 (0.46) | 0.9906 |  |
| 12 months vs baseline | -0.83 (0.72) | 0.9439 | 0.41 (0.8) | 0.9996 |  | 12 months | 0.13 (0.5) | 1.0000 |  |
| SF-12 MCS |  |  |  |  |  |  |  |  | 0.6307 |
|  |  |  |  |  |  | Baseline | 0.73 (0.9) | 0.9921 |  |
| 1 months vs baseline | -1.53 (0.58) | 0.1497 | -0.43 (0.72) | 0.9989 |  | 1 month | 0.1 (0.96) | 1.0000 |  |
| 3 months vs baseline | -1.06 (0.62) | 0.6860 | -0.54 (0.79) | 0.9972 |  | 3 months | -0.42 (0.45) | 0.9956 |  |
| 6 months vs baseline | -0.11 (0.61) | 1.0000 | 0.14 (0.83) | 1.0000 |  | 6 months | -0.47 (0.46) | 0.9906 |  |
| 12 months vs baseline | 0.57 (0.66) | 0.9885 | -1.16 (0.91) | 0.9100 |  | 12 months | 0.13 (0.5) | 1.0000 |  |

*Abbreviations:* ENI, ElectiveNodalIrradiation; UCLA-PCI, UCLA Prostate Cancer Index; UF, UrinaryFunction; UB, UrinaryBother; BF, BowelFunction; BB, BowelBother; SF, SexualFunction; SB, SexualBother; SF-12, Short Form survey 12; PCS, Physical Component Summary; MCS, Mental Component Summary.

Estimated mean differences and 95% CI from mixed-model repeated measures analyses, adjusted for score at diagnosis, age at diagnosis, presence of diabetes mellitus, number of comorbidities according to CIRS, risk according to NCCN, aim of the RT (exclusive, adjuvant, salvage), RT method (IGRT, no IGRT), RT technique (IMRT (step and shoot or 3D-CRT), IMRT (volumetric)), and ADT.

**Supplementary Table 3b.** Comparison of variation of UCLA-PCI and SF-12 scores over time, for ENI vs no ENI groups (numbers indicate estimated mean difference and 95% CI), considering **only patients not submitted to prostatectomy**

|  | **Estimated differences**  ***within groups*** | | | |  | **Estimated differences**  ***between groups*** | | | **p-value interaction**  **group*time** |
| --- | --- | --- | --- | --- | --- | --- | --- | --- | --- |
|  | **ENI** | **p-value** | **No ENI** | **p-value** |  |  | **ENI vs no ENI** | **p-value** |  |
| UCLA-PCI UF |  |  |  |  |  |  |  |  | 0.6611 |
|  |  |  |  |  |  | Baseline | 0.46 (1.52) | 1.0000 |  |
| 1 months vs baseline | -3.68 (1.25) | 0.0673 | -2.08 (0.90) | 0.2921 |  | 1 month | -0.25 (1.59) | 1.0000 |  |
| 3 months vs baseline | -2.80 (1.28) | 0.3639 | -2.73 (1.08) | 0.1862 |  | 3 months | -0.42 (0.45) | 0.9956 |  |
| 6 months vs baseline | -0.65 (0.88) | 0.9957 | -3.26 (1.08) | 0.0529 |  | 6 months | -0.47 (0.46) | 0.9906 |  |
| 12 months vs baseline | -1.18 (0.98) | 0.9320 | -2.55 (1.53) | 0.7121 |  | 12 months | 0.13 (0.50) | 1.0000 |  |
| UCLA-PCI UB |  |  |  |  |  |  |  |  | 0.6141 |
|  |  |  |  |  |  | Baseline | 0.03 (2.38) | 1.0000 |  |
| 1 months vs baseline | -11.26 (1.98) | <0.0001 | -6.31 (1.51) | 0.0009 |  | 1 month | -4.44 (2.27) | 0.5142 |  |
| 3 months vs baseline | -10.78 (1.98) | <0.0001 | -8.05 (1.71) | <0.0001 |  | 3 months | -0.42 (0.45) | 0.9956 |  |
| 6 months vs baseline | -1.74 (1.43) | 0.9275 | -9.35 (1.67) | <0.0001 |  | 6 months | -0.47 (0.46) | 0.9906 |  |
| 12 months vs baseline | -3.04 (1.50) | 0.4624 | -6.34 (2.33) | 0.1174 |  | 12 months | 0.13 (0.50) | 1.0000 |  |
| UCLA-PCI BF |  |  |  |  |  |  |  |  | 0.5366 |
|  |  |  |  |  |  | Baseline | 0.61 (1.74) | 1.0000 |  |
| 1 months vs baseline | -4.64 (1.53) | 0.0516 | -5.48 (1.19) | 0.0001 |  | 1 month | 0.53 (1.58) | 1.0000 |  |
| 3 months vs baseline | -5.57 (1.58) | 0.0107 | -5.99 (1.33) | 0.0002 |  | 3 months | -0.42 (0.45) | 0.9956 |  |
| 6 months vs baseline | -0.51 (1.05) | 0.9997 | -5.29 (1.34) | 0.0022 |  | 6 months | -0.47 (0.46) | 0.9906 |  |
| 12 months vs baseline | 0.19 (1.19) | 1.0000 | -6.09 (1.66) | 0.0065 |  | 12 months | 0.13 (0.50) | 1.0000 |  |
| UCLA-PCI BB |  |  |  |  |  |  |  |  | 0.4631 |
|  |  |  |  |  |  | Baseline | 0.81 (2.64) | 1.0000 |  |
| 1 months vs baseline | -1.76 (2.15) | 0.9919 | -3.07 (1.73) | 0.6392 |  | 1 month | -0.57 (3.09) | 1.0000 |  |
| 3 months vs baseline | -4.45 (2.55) | 0.6568 | -2.08 (1.86) | 0.9529 |  | 3 months | -0.42 (0.45) | 0.9956 |  |
| 6 months vs baseline | 0.98 (1.72) | 0.9992 | -2.73 (2.17) | 0.9129 |  | 6 months | -0.47 (0.46) | 0.9906 |  |
| 12 months vs baseline | 0.33 (2.15) | 1.0000 | -3.88 (2.75) | 0.8538 |  | 12 months | 0.13 (0.50) | 1.0000 |  |
| UCLA-PCI SF |  |  |  |  |  |  |  |  | 0.1904 |
|  |  |  |  |  |  | Baseline | 0.16 (0.07) | 0.3863 |  |
| 1 months vs baseline | 1.01 (0.07) | <0.0001 | 0.31 (0.07) | 0.0002 |  | 1 month | -1.54 (0.07) | <0.0001 |  |
| 3 months vs baseline | -1.39 (0.09) | <0.0001 | 1.18 (0.06) | <0.0001 |  | 3 months | -0.42 (0.45) | 0.9956 |  |
| 6 months vs baseline | 0.87 (0.04) | <0.0001 | -1.19 (0.08) | <0.0001 |  | 6 months | -0.47 (0.46) | 0.9906 |  |
| 12 months vs baseline | -1.50 (0.06) | <0.0001 | 0.15 (0.07) | 0.3000 |  | 12 months | 0.13 (0.50) | 1.0000 |  |
| UCLA-PCI SB |  |  |  |  |  |  |  |  | 0.8789 |
|  |  |  |  |  |  | Baseline | 0.38 (2.28) | 1.0000 |  |
| 1 months vs baseline | -6.38 (1.93) | 0.0225 | -6.06 (1.50) | 0.0015 |  | 1 month | 0.67 (2.11) | 1.0000 |  |
| 3 months vs baseline | -5.77 (2.03) | 0.0877 | -6.83 (1.67) | 0.0013 |  | 3 months | -0.42 (0.45) | 0.9956 |  |
| 6 months vs baseline | -0.77 (1.30) | 0.9989 | -5.80 (1.73) | 0.0193 |  | 6 months | -0.47 (0.46) | 0.9906 |  |
| 12 months vs baseline | 0.26 (1.50) | 1.0000 | -6.43 (2.18) | 0.0651 |  | 12 months | 0.13 (0.50) | 1.0000 |  |
| SF-12 PCS |  |  |  |  |  |  |  |  | 0.9666 |
|  |  |  |  |  |  | Baseline | -0.79 (0.66) | 0.9330 |  |
| 1 months vs baseline | -0.31 (0.55) | 0.9992 | -0.35 (0.41) | 0.9897 |  | 1 month | -0.42 (0.73) | 0.9991 |  |
| 3 months vs baseline | 0.01 (0.60) | 1.0000 | -0.40 (0.47) | 0.9888 |  | 3 months | -0.42 (0.45) | 0.9956 |  |
| 6 months vs baseline | -0.05 (0.38) | 1.0000 | -0.28 (0.50) | 0.9992 |  | 6 months | -0.47 (0.46) | 0.9906 |  |
| 12 months vs baseline | 0.07 (0.45) | 1.0000 | 0.44 (0.66) | 0.9979 |  | 12 months | 0.13 (0.50) | 1.0000 |  |
| SF-12 MCS |  |  |  |  |  |  |  |  | 0.2565 |
|  |  |  |  |  |  | Baseline | -2.21 (0.71) | 0.0438 |  |
| 1 months vs baseline | -0.36 (0.60) | 0.9988 | -0.77 (0.47) | 0.7212 |  | 1 month | -1.17 (0.79) | 0.8144 |  |
| 3 months vs baseline | 0.27 (0.68) | 0.9999 | -0.30 (0.51) | 0.9990 |  | 3 months | -0.42 (0.45) | 0.9956 |  |
| 6 months vs baseline | 0.47 (0.43) | 0.9619 | 0.33 (0.57) | 0.9991 |  | 6 months | -0.47 (0.46) | 0.9906 |  |
| 12 months vs baseline | 1.10 (0.50) | 0.3696 | 1.44 (0.73) | 0.5040 |  | 12 months | 0.13 (0.50) | 1.0000 |  |

*Abbreviations:* ENI, ElectiveNodalIrradiation; UCLA-PCI, UCLA Prostate Cancer Index; UF, UrinaryFunction; UB, UrinaryBother; BF, BowelFunction; BB, BowelBother; SF, SexualFunction; SB, SexualBother; SF-12, Short Form survey 12; PCS, Physical Component Summary; MCS, Mental Component Summary.

Estimated mean differences and 95% CI from mixed-model repeated measures analyses, adjusted for score at diagnosis, age at diagnosis, presence of diabetes mellitus, number of comorbidities according to CIRS, risk according to NCCN, aim of the RT (exclusive, adjuvant, salvage), RT method (IGRT, no IGRT), RT technique (IMRT (step and shoot or 3D-CRT), IMRT (volumetric)), and ADT.

**Supplementary Table 4.a**Rectal toxicity *at 12 months*, by RT features

|  | **G0** | **G1** | **G2** | **G3** | **G4** | **p-value** |
| --- | --- | --- | --- | --- | --- | --- |
| Aim of the RT, n (%)  Exclusive  Adjuvant  Salvage | 435 (89.3)  211 (92.5)  43 (91.5) | 37 (7.6)  10 (4.4)  3 (6.4) | 13 (2.7)  6 (2.7)  0 (0.0) | 2 (0.4)  1 (0.4)  1 (2.1) | 0 (0.0)  0 (0.0)  0 (0.0) | 0.4775 |
| RT method, n (%)  IGRT  no IGRT | 612 (91.1)  77 (85.6) | 42 (6.3)  8 (8.9) | 16 (2.4)  3 (3.3) | 2 (0.2)  2 (2.2) | 0 (0.0)  0 (0.0) | 0.0377 |
| RT technique, n (%)  IMRT (step and shoot) or 3D-CRT  IMRT (volumetric)  SBRT | 129 (88.4)  552 (90.8)  8 (100.0) | 11 (7.5)  39 (6.4)  0 (0.0) | 4 (2.7)  15 (2.5)  0 (0.0) | 2 (1.4)  2 (0.3)  0 (0.0) | 0 (0.0)  0 (0.0)  0 (0.0) | 0.1918 |
| ENI, n (%)  Yes  No | 332 (92.5)  357 (88.6) | 20 (5.6)  30 (7.4) | 6 (1.6)  13 (3.2) | 1 (0.3)  3 (0.8) | 0 (0.0)  0 (0.0) | 0.0469 |
| ENI and RT method, n (%)  ENI and IGRT  ENI, no IGRT  No ENI and IGRT  No ENI, no IGRT | 292 (92.1)  40 (95.2)  320 (90.1)  37 (77.1) | 18 (5.7)  2 (4.8)  24 (6.8)  6 (12.5) | 6 (1.9)  0 (0.0)  10 (2.8)  3 (6.3) | 1 (0.3)  0 (0.0)  1 (0.3)  2 (4.1) | 0 (0.0)  0 (0.0)  0 (0.0)  0 (0.0) | 0.0115 |
| ENI and RT technique, n (%) *  ENI and IMRT (volumetric)  ENI and (IMRT (step and shoot) or 3D-CRT)  No ENI and IMRT (volumetric)  No ENI and (IMRT (step and shoot) or 3D-CRT) | 274 (92.3)  57 (93.4)  278 (89.4)  72 (84.7) | 16 (5.4)  4 (6.6)  23 (7.4)  7 (8.2) | 6 (2.0)  0 (0.0)  9 (2.9)  4 (4.7) | 1 (0.3)  0 (0.0)  1 (0.3)  2 (2.4) | 0 (0.0)  0 (0.0)  0 (0.0)  0 (0.0) | 0.0220 |

*Abbreviations:* ENI, ElectiveNodalIrradiation;IGRT, Image-GuidedRadiation Therapy; IMRT, Intensity-ModulatedRadiation Therapy;RT, Radiotherapy; 3D-CRT, three-dimensionalconformalradiotherapy; SBRT, Stereotactic body radiotherapy

* Missing values for 8 patients

**Supplementary Table 4b.** Generalized Estimating Equations for ordinal data related to rectal toxicity, by RT features

|  | **Rectal Toxicity** | | |
| --- | --- | --- | --- |
|  | **OR** | **95% CI** | **p-value** |
| Aim of the RT, Adjuvant or salvage vs exclusive | 0.81 | 0.57-1.16 | 0.2492 |
| RT modality, IGRT vs no IGRT | 0.58 | 0.40-0.85 | 0.0049 |
| RT technique, IMRT (volumetric) vs (IMRT (step and shoot) or 3D-CRT) | 1.09 | 0.79-1.51 | 0.5884 |
| ENI vs no ENI | 1.33 | 0.98-1.81 | 0.9283 |
| ENI and RT method  (no ENI and IGRT) vs (no ENI, no IGRT)  (ENI, no IGRT) vs (no ENI, no IGRT)  (ENI and IGRT) vs (no ENI, no IGRT)  (ENI, no IGRT) vs (no ENI and IGRT)  (ENI and IGRT) vs (no ENI and IGRT)  (ENI and IGRT) vs (ENI, no IGRT) | 0.47  0.58  0.50  1.24  1.06  0.85 | 0.27-0.82  0.27-1.26  0.28-0.90  0.68-2.28  0.77-1.46  0.46-1.58 | 0.0074  0.1720  0.0203  0.4812  0.7230  0.6114 |
| ENI and RT technique  (no ENI andIMRT (volumetric)) vs (no ENI and (IMRT (step and shoot) or 3D-CRT))  (ENI and IMRT (step and shoot) or 3D-CRT) vs (no ENI and (IMRT (step and shoot) or 3D-CRT)  (ENI and IMRT (volumetric) vs (no ENI and (IMRT (step and shoot) or 3D-CRT)  (ENI and IMRT (step and shoot) or 3D-CRT) vs (no ENI and (IMRT (volumetric))  (ENI and IMRT (volumetric) vs (no ENI and (IMRT (volumetric))  (ENI and IMRT (step and shoot) or 3D-CRT) vs (ENI and (IMRT (volumetric)) | 0.99  0.77  1.01  0.78  1.02  0.33 | 0.63-1.55  0.43-1.40  0.65-1.58  0.46-1.32  0.72-1.44  0.80-2.16 | 0.9729  0.3916  0.9544  0.3490  0.9062  0.2842 |

*Abbreviations:* ENI, ElectiveNodalIrradiation; IGRT, Image-GuidedRadiation Therapy; IMRT, Intensity-ModulatedRadiation Therapy; RT, Radiotherapy; 3D-CRT, three-dimensionalconformalradiotherapy; SBRT, Stereotactic body radiotherapy

GEE adjusted for age at diagnosis, presence of diabetes mellitus, number of comorbidities according to CIRS, risk according to NCCN, aim of the RT (exclusive, adjuvant, salvage), RT method (IGRT, no IGRT), RT technique (IMRT (step and shoot or 3D-CRT), IMRT (volumetric)

**Supplementary Table 5.a** Urinary toxicity *at 12 months* by RT features

|  | **G0** | **G1** | **G2** | **G3** | **G4** | **p-value** |
| --- | --- | --- | --- | --- | --- | --- |
| Aim of the RT, n (%)  Exclusive  Adjuvant  Salvage | 383 (78.6)  169 (74.2)  37 (78.7) | 84 (17.3)  47 (20.6)  6 (12.8) | 18 (3.7)  11 (4.8)  3 (6.4) | 1 (0.2)  0 (0.0)  1 (2.1) | 1 (0.2)  1 (0.4)  0 (0.0) | 0.2092 |
| RT method, n (%)  IGRT  no IGRT | 509 (75.7)  80 (88.9) | 131 (19.5)  6 (6.7) | 28 (4.2)  4 (4.4) | 2 (0.3)  0 (0.0) | 2 (0.3)  0 (0.0) | 0.0270 |
| RT technique, n (%)  IMRT (step and shoot) or 3D-CRT  IMRT (volumetric)  SBRT | 118 (80.9)  463 (76.2)  8 (100.0) | 23 (18.7)  114 (18.8)  0 (0.0) | 5 (3.4)  27 (4.4)  0 (0.0) | 0 (0.0)  2 (0.3)  0 (0.0) | 0 (0.0)  2 (0.3)  0 (0.0) | 0.2676 |
| ENI, n (%)  Yes  No | 276 (76.9)  313 (77.7) | 67 (18.8) 70 (17.4) | 14 (3.9)  18 (4.5) | 1 (0.2)  1 (0.2) | 1 (0.2)  1 (0.2) | 0.9292 |
| ENI and RT method, n (%)  ENI and IGRT  ENI, no IGRT  No ENI and IGRT  No ENI, no IGRT | 239 (75.4)  37 (88.1)  270 (76.1)  43 (89.6) | 63 (19.9)  4 (9.5)  68 (19.2)  2 (4.1) | 13 (4.1)  1 (2.4)  15 (4.1)  3 (6.3) | 1 (0.3)  0 (0.0)  1 (0.3)  0 (0.0) | 1 (0.3)  0 (0.0)  1 (0.3)  0 (0.0) | 0.4447 |
| ENI and RT technique, n (%)  ENI and IMRT (volumetric)  ENI and (IMRT (step and shoot) or 3D-CRT)  No ENI and IMRT (volumetric)  No ENI and (IMRT (step and shoot) or 3D-CRT) | 224 (75.5)  51 (83.6)  239 (76.9)  67 (78.8) | 59 (19.9)  8 (13.1)  55 (17.7)  15 (17.7) | 12 (4.0)  2 (3.3)  15 (4.8)  3 (3.5) | 1 (0.3)  0 (0.0)  1 (0.3)  0 (0.0) | 1 (0.3)  0 (0.0)  1 (0.3)  0 (0.0) | 0.6274 |

*Abbreviations:* ENI, ElectiveNodalIrradiation; IGRT, Image-GuidedRadiation Therapy; IMRT, Intensity-ModulatedRadiation Therapy; RT, Radiotherapy; 3D-CRT, three-dimensionalconformalradiotherapy; SBRT, Stereotactic body radiotherapy

* Missing values for 8 patients

**Supplementary Table 5.b** Generalized Estimating Equations for ordinal data related to urinary toxicity, by RT features

|  | **Urinary Toxicity** | | |
| --- | --- | --- | --- |
|  | **OR** | **95% CI** | **p-value** |
| Aim of the RT, Adjuvant or Salvage vs Exclusive | 1.31 | 1.01-1.69 | 0.0435 |
| RT modality, IGRT vs no IGRT | 1.41 | 0.98-2.01 | 0.0604 |
| RT technique, IMRT (volumetric) vs ((IMRT (step and shoot) or 3D-CRT)) | 1.03 | 0.77-1.38 | 0.8524 |
| ENI vs no ENI | 0.97 | 0.77-1.22 | 0.6583 |
| ENI and RT method  (No ENI and IGRT) vs (No ENI, no IGRT)  (ENI, no IGRT) vs (No ENI, no IGRT)  (ENI and IGRT) vs (No ENI, no IGRT)  (ENI, no IGRT) vs (No ENI and IGRT)  (ENI and IGRT) vs (No ENI and IGRT)  (ENI and IGRT) vs (ENI, no IGRT) | 1.43  0.99  1.42  0.70  0.99  1.43 | 0.85-2.39  0.50-1.96  0.83-2.43  0.43-1.14  0.78-1.27  0.88-2.35 | 0.1800  0.9826  0.1979  0.1494  0.9882  0.1515 |
| ENI and RT technique  (no ENI and IMRT (volumetric)) vs (no ENI and (IMRT (step and shoot) or 3D-CRT))  (ENI and IMRT (step and shoot) or 3D-CRT) vs (no ENI and (IMRT (step and shoot) or 3D-CRT)  (ENI and IMRT (volumetric) vs (no ENI and (IMRT (step and shoot) or 3D-CRT)  (ENI and IMRT (step and shoot) or 3D-CRT) vs (no ENI and (IMRT (volumetric))  (ENI andIMRT (volumetric) vs (no ENI and (IMRT (volumetric))  (ENI and IMRT (volumetric)) vs (ENI and (IMRT (step and shoot) or 3D-CRT)) | 0.73  0.53  0.83  0.73  1.15  1.57 | 0.50-1.05  0.31-0.90  0.59-1.18  0.45-1.18  0.89-1.48  0.99-2.48 | 0.0908  0.092  0.3040  0.1998  0.2889  0.0507 |

*Abbreviations:* ENI, ElectiveNodalIrradiation; IGRT, Image-GuidedRadiation Therapy; IMRT, Intensity-ModulatedRadiation Therapy; RT, Radiotherapy; 3D-CRT, three-dimensionalconformalradiotherapy; SBRT, Stereotactic body radiotherapy

GEE adjusted for age at diagnosis, presence of diabetes mellitus, number of comorbidities according to CIRS, risk according to NCCN, aim of the RT (exclusive, adjuvant, salvage), RT method (IGRT, no IGRT), RT technique (IMRT (step and shoot or 3D-CRT), IMRT (volumetric)

**Supplementary Table 6.** Bowel toxicity *at 12 months* by RT features

|  | **G0** | **G1** | **G2** | **G3** | **G4** | **p-value** |
| --- | --- | --- | --- | --- | --- | --- |
| Aim of the RT, n (%)  Exclusive  Adjuvant  Salvage | 478 (98.2)  217 (95.2)  45 (95.8) | 7 (1.4)  8 (3.5)  1 (2.1) | 2 (0.4)  3 (1.3)  1 (2.1) | 0 (0.0)  0 (0.0)  0 (0.0) | 0 (0.0)  0 (0.0)  0 (0.0) | 0.0310 |
| RT method, n (%)  IGRT  no IGRT | 658 (97.9)  82 (91.2) | 12 (1.8)  4 (4.4) | 2 (0.3)  4 (4.4) | 0 (0.0)  0 (0.0) | 0 (0.0)  0 (0.0) | <0.0001 |
| RT technique, n (%)  IMRT (step and shoot) or 3D-CRT  IMRT (volumetric)  SBRT | 138 (94.6)  594 (97.7)  8 (100.0) | 4 (2.7)  12 (2.0)  0 (0.0) | 4 (2.7)  2 (0.3)  0 (0.0) | 0 (0.0)  0 (0.0)  0 (0.0) | 0 (0.0)  0 (0.0)  0 (0.0) | 0.0066 |
| ENI, n (%)  Yes  No | 350 (97.5) 390 (96.8) | 7 (2.0)  9 (2.2) | 2 (0.5)  4 (1.0) | 0 (0.0)  0 (0.0) | 0 (0.0)  0 (0.0) | 0.4822 |
| ENI and RT method, n (%)  ENI and IGRT  ENI, no IGRT  No ENI and IGRT  No ENI, no IGRT | 309 (97.5)  41 (97.6)  349 (98.3)  41 (85.4) | 6 (1.9)  1 (2.4)  6 (1.7)  3 (6.3) | 2 (0.6)  0 (0.0)  0 (0.0)  4 (8.3) | 0 (0.0)  0 (0.0)  0 (0.0)  0 (0.0) | 0 (0.0)  0 (0.0)  0 (0.0)  0 (0.0) | 0.0462 |
| ENI and RT technique, n (%)  ENI and IMRT (volumetric)  ENI and (IMRT (step and shoot) or 3D-CRT)  No ENI and IMRT (volumetric)  No ENI and (IMRT (step and shoot) or 3D-CRT) | 290 (97.6)  59 (96.7)  304 (97.7)  79 (92.9) | 5 (1.7)  2 (3.3)  7 (2.3)  2 (2.4) | 2 (0.7)  0 (0.0)  0 (0.0)  4 (4.7) | 0 (0.0)  0 (0.0)  0 (0.0)  0 (0.0) | 0 (0.0)  0 (0.0)  0 (0.0)  0 (0.0) | 0.1023 |

*Abbreviations:* ENI, ElectiveNodalIrradiation; IGRT, Image-GuidedRadiation Therapy; IMRT, Intensity-ModulatedRadiation Therapy; RT, Radiotherapy; 3D-CRT, three-dimensionalconformalradiotherapy; SBRT, Stereotactic body radiotherapy

* Missing values for 8 patients

**Supplementary Table 6a.**Generalized Estimating Equations for ordinal data related to bowel toxicity, by RT features

|  | **Bowel Toxicity** | | |
| --- | --- | --- | --- |
|  | **OR** | **95% CI** | **p-value** |
| Aim of the RT, Adjuvant or Salvage vs Exclusive | 1.38 | 0.81-2.39 | 0.2444 |
| RT modality, IGRT vs no IGRT | 0.33 | 0.19-0.56 | <0.0001 |
| RT technique, IMRT (volumetric) vs ((IMRT (step and shoot) or 3D-CRT)) | 0.97 | 0.60-1.58 | 0.9066 |
| ENI vs no ENI | 1.20 | 0.73-1.97 | 0.4767 |
| ENI and RT method  (No ENI and IGRT) vs (No ENI, no IGRT)  (ENI, no IGRT) vs (No ENI, no IGRT)  (ENI and IGRT) vs (No ENI, no IGRT)  (ENI, no IGRT) vs (No ENI and IGRT)  (ENI and IGRT) vs (No ENI and IGRT)  (ENI and IGRT) vs (ENI, no IGRT) | 0.18  0.40  0.31  2.22  1.73  0.78 | 0.08-0.39  0.14-1.17  0.14-0.71  0.92-5.34  1.01-2.98  0.33-1.87 | <0.0001  0.0940  0.0051  0.0757  0.0481  0.5764 |
| ENI and RT technique  (no ENI and IMRT (volumetric)) vs (no ENI and (IMRT (step and shoot) or 3D-CRT))  (ENI and IMRT (step and shoot) or 3D-CRT) vs (no ENI and (IMRT (step and shoot) or 3D-CRT)  (ENI and IMRT (volumetric) vs (no ENI and (IMRT (step and shoot) or 3D-CRT)  (ENI and IMRT (step and shoot) or 3D-CRT) vs (no ENI and (IMRT (volumetric))  (ENI and IMRT (volumetric) vs (no ENI and (IMRT (volumetric))  (ENI and IMRT (volumetric)) vs (ENI and (IMRT (step and shoot) or 3D-CRT)) | 0.60  0.56  0.98  0.95  1.65  1.75 | 0.31-1.13  0.21-1.54  0.52-1.85  0.36-2.51  0.94-2.90  0.70-4.36 | 0.1120  0.2634  0.9619  0.9136  0.0799  0.2336 |

*Abbreviations:* ENI, ElectiveNodalIrradiation; IGRT, Image-GuidedRadiation Therapy; IMRT, Intensity-ModulatedRadiation Therapy; RT, Radiotherapy; 3D-CRT, three-dimensionalconformalradiotherapy; SBRT, Stereotactic body radiotherapy

GEE adjusted for age at diagnosis, presence of diabetes mellitus, number of comorbidities according to CIRS, risk according to NCCN, aim of the RT (exclusive, adjuvant, salvage), RT method (IGRT, no IGRT), RT technique (IMRT (step and shoot or 3D-CRT), IMRT (volumetric)

**Figure 4.** Rectal toxicity, by time and RT features


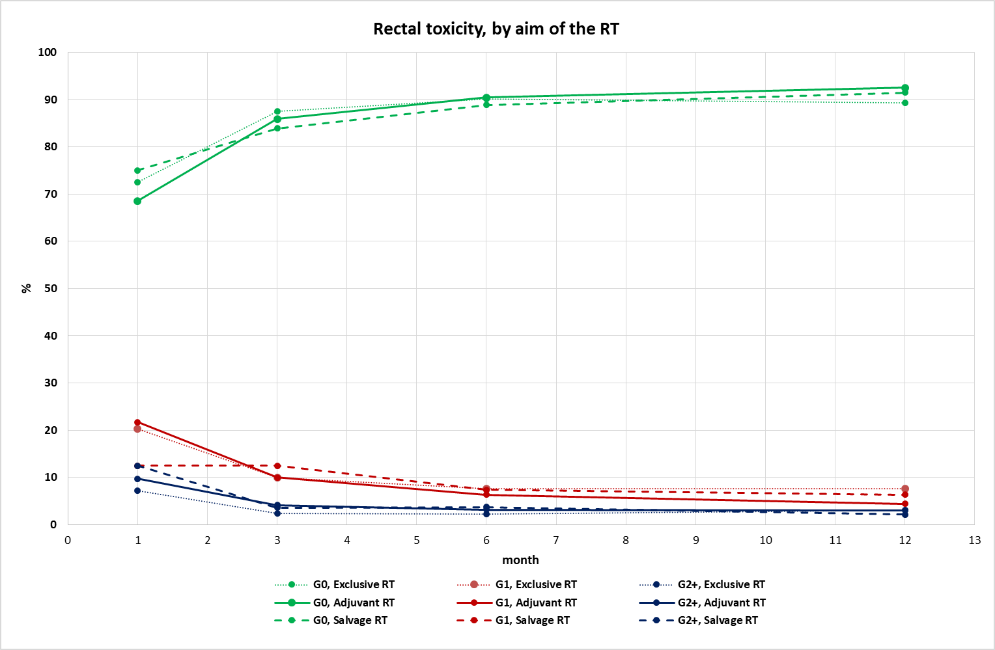


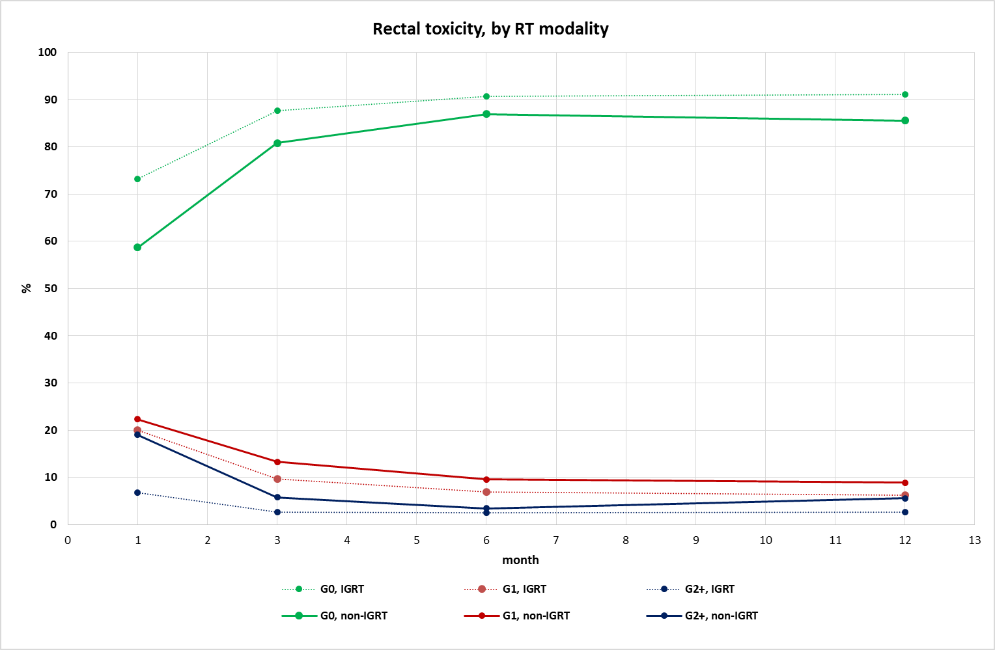


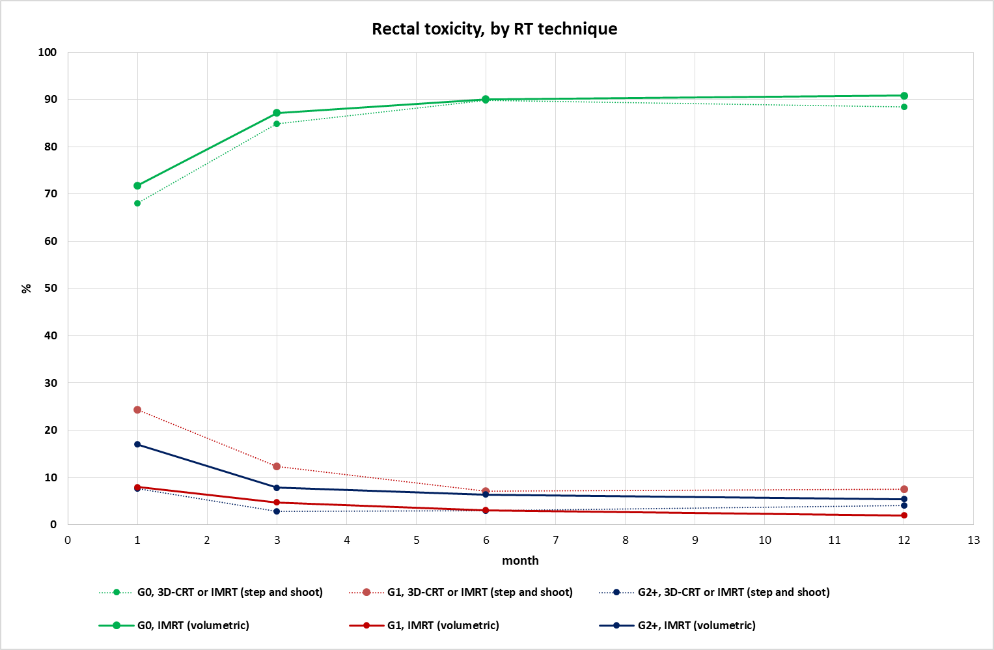


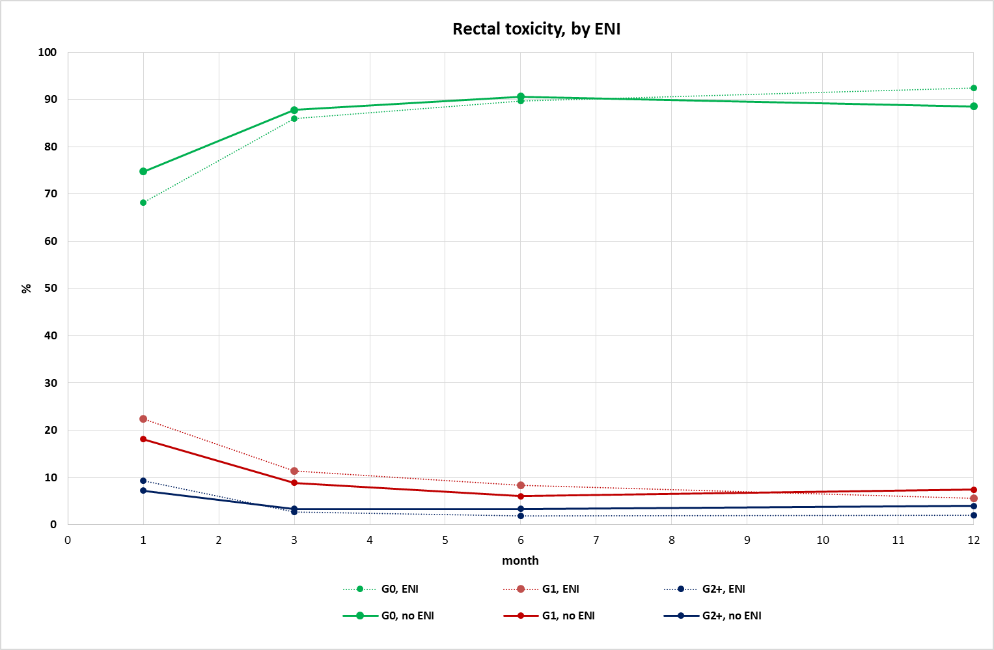


**Figure 5.** Urinary toxicity, by time and RT features


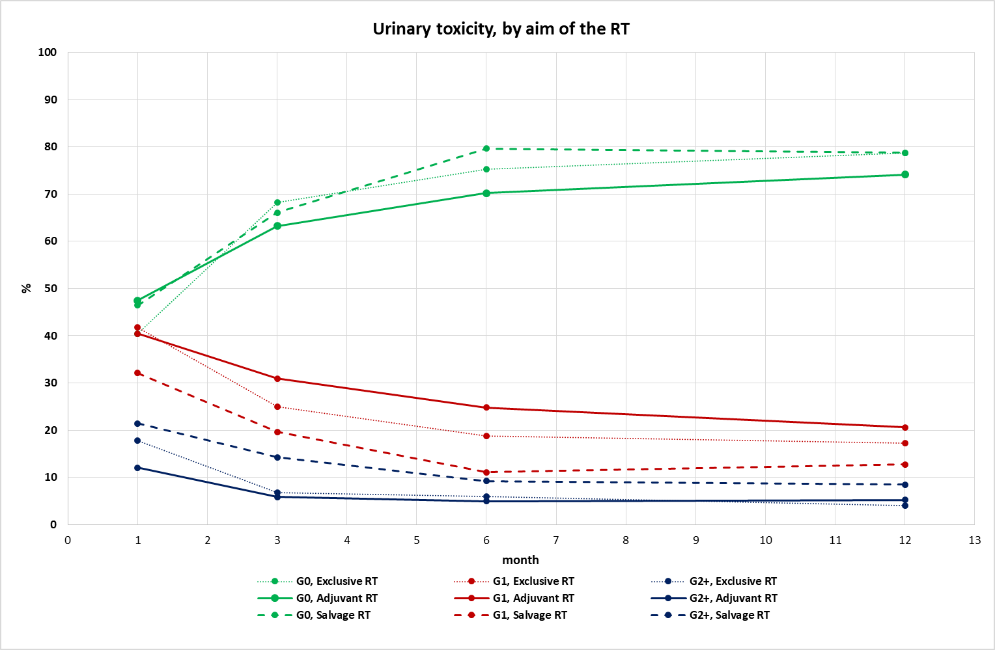


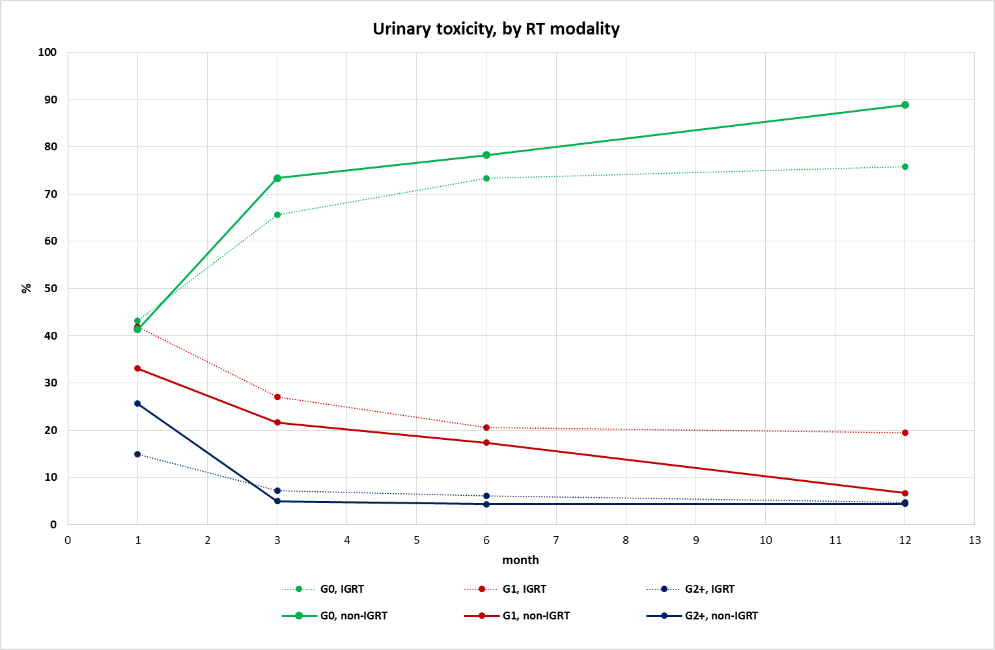


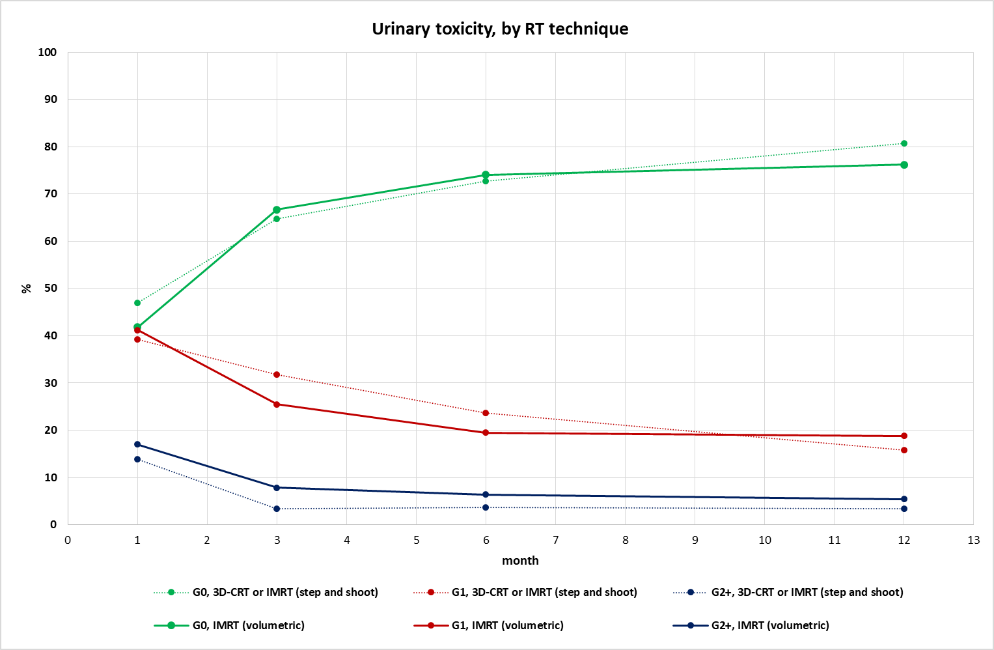


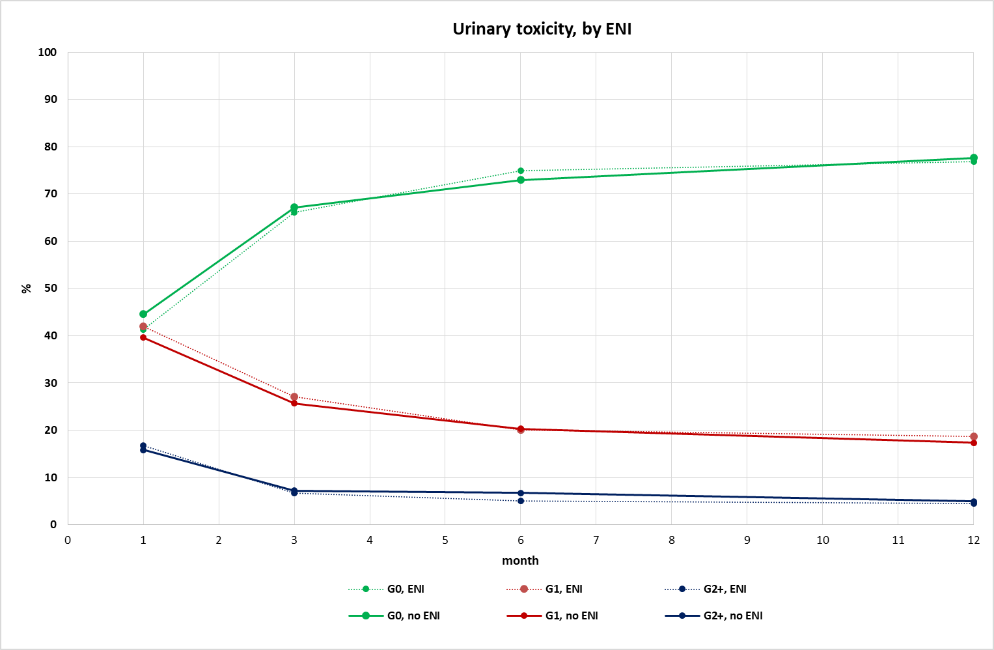


**Figure 6.** Bowel toxicity, by time and RT features


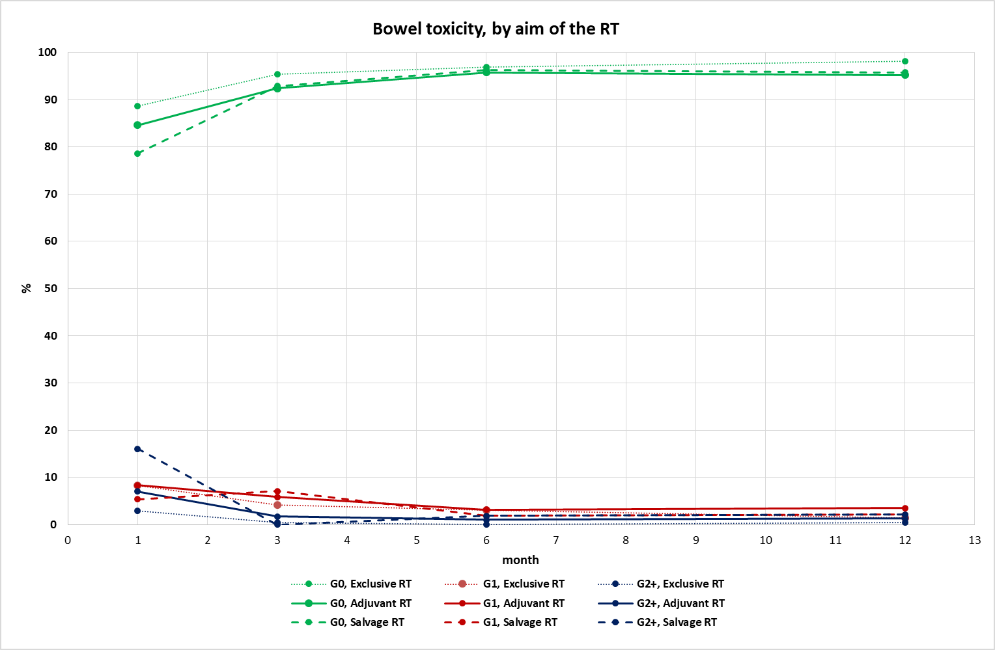


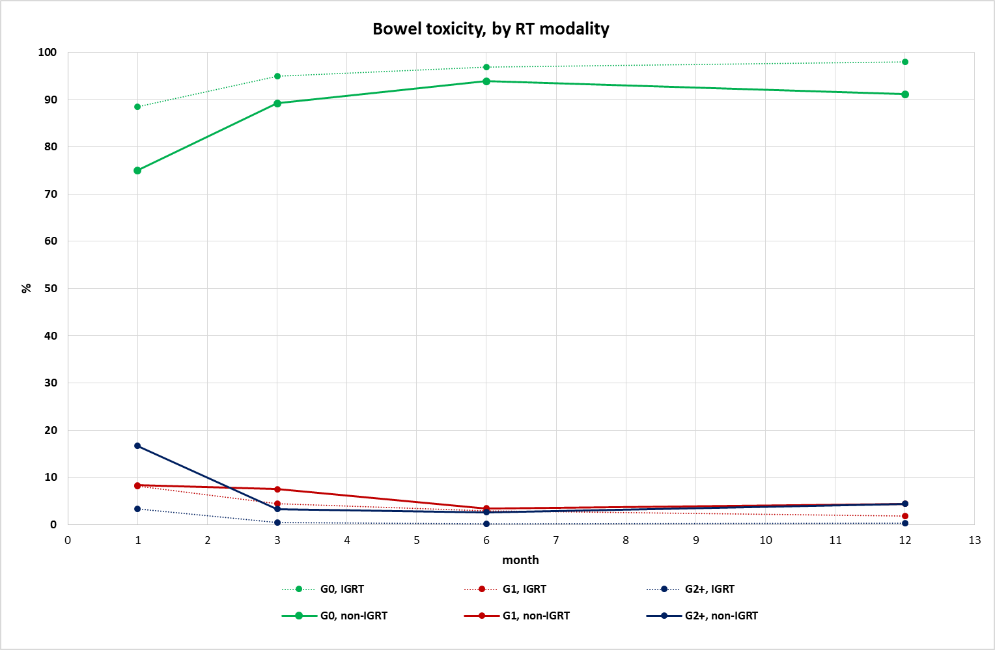


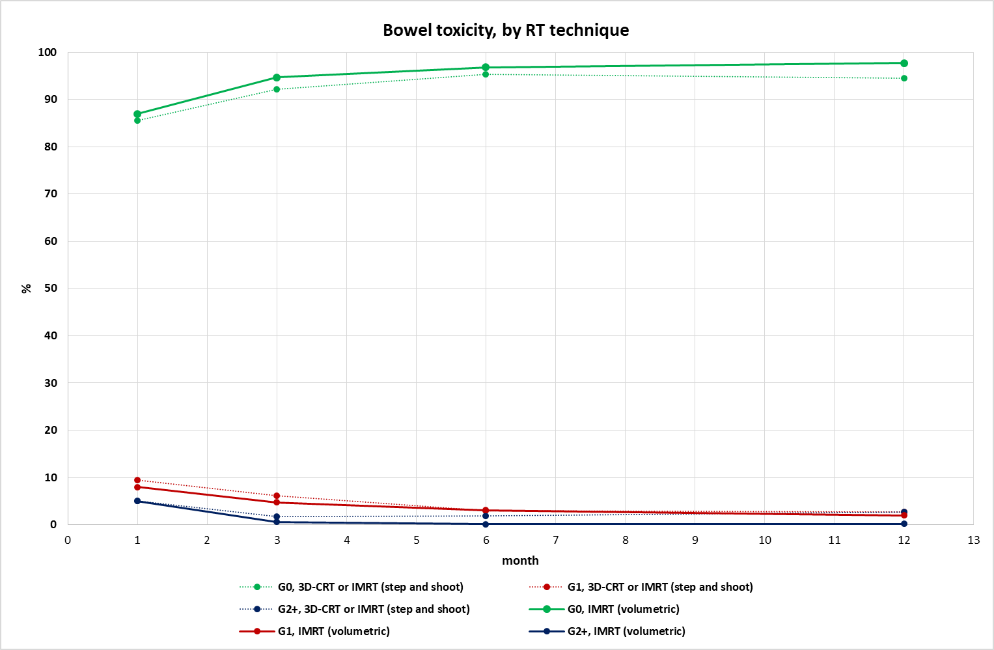


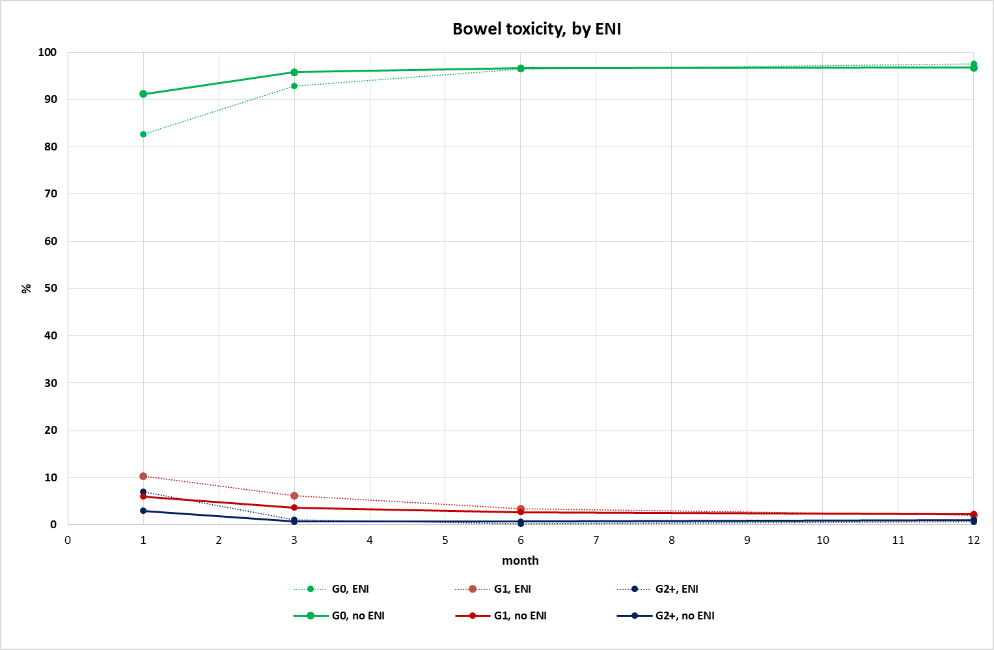

Supplement: Supplementary file 1 [file DataSheet_1.docx]
